# Genome of *Epinotia aporema* granulovirus (EpapGV), a polyorganotropic fast killing betabaculovirus with a novel thymidylate kinase gene

**DOI:** 10.1186/1471-2164-13-548

**Published:** 2012-10-11

**Authors:** María Leticia Ferrelli, Ricardo Salvador, Marina Elizabeth Biedma, Marcelo Facundo Berretta, Santiago Haase, Alicia Sciocco-Cap, Pablo Daniel Ghiringhelli, Víctor Romanowski

**Affiliations:** 1 Instituto de Biotecnología y Biología Molecular, Facultad de Ciencias Exactas, Universidad Nacional de La Plata, CONICET, La Plata, Argentina; 2 Instituto de Microbiología y Zoología Agrícola, INTA, Castelar, Argentina; 3 Laboratorio de Ingeniería Genética y Biología Celular y Molecular - Area Virosis de Insectos, Departamento de Ciencia y Tecnología, Universidad Nacional de Quilmes, Bernal, Argentina; 4 Present address: INSERM U748, Institut de Virologie, Faculté de Médecine, Université de Strasbourg, 3 rue Koeberlé, 67000, Strasbourg, France

## Abstract

**Background:**

*Epinotia aporema* (Lepidoptera: Tortricidae) is an important pest of legume crops in South America. *Epinotia aporema* granulovirus (EpapGV) is a baculovirus that causes a polyorganotropic infection in the host larva. Its high pathogenicity and host specificity make EpapGV an excellent candidate to be used as a biological control agent.

**Results:**

The genome of *Epinotia aporema* granulovirus (EpapGV) was sequenced and analyzed. Its circular double-stranded DNA genome is 119,082 bp in length and codes for 133 putative genes. It contains the 31 baculovirus core genes and a set of 19 genes that are GV exclusive. Seventeen ORFs were unique to EpapGV in comparison with other baculoviruses. Of these, 16 found no homologues in GenBank, and one encoded a thymidylate kinase. Analysis of nucleotide sequence repeats revealed the presence of 16 homologous regions (*hrs*) interspersed throughout the genome. Each *hr* was characterized by the presence of 1 to 3 clustered imperfect palindromes which are similar to previously described palindromes of tortricid-specific GVs. Also, one of the *hrs* (*hr4*) has flanking sequences suggestive of a putative non*-hr* ori. Interestingly, two more complex *hrs* were found in opposite loci, dividing the circular dsDNA genome in two halves. Gene synteny maps showed the great colinearity of sequenced GVs, being EpapGV the most dissimilar as it has a 20 kb-long gene block inversion. Phylogenetic study performed with 31 core genes of 58 baculoviral genomes suggests that EpapGV is the baculovirus isolate closest to the putative common ancestor of tortricid specific betabaculoviruses.

**Conclusions:**

This study, along with previous characterization of EpapGV infection, is useful for the better understanding of the pathology caused by this virus and its potential utilization as a bioinsecticide.

## Background

Baculoviruses (family *Baculoviridae*) are rod-shaped, enveloped, insect-specific viruses with double-stranded, circular DNA genomes ranging in size from 80 to 180 kb [1]. The family *Baculoviridae* is subdivided into four genera: *Alphabaculovirus* (lepidopteran-specific nucleopolyhedrovirus, NPVs), *Betabaculovirus* (lepidopteran-specific granulovirus, GVs), *Gammabaculovirus* (hymenopteran-specific NPVs) and *Deltabaculovirus* (dipteran-specific NPV) [2,3]. GVs have been isolated only from insects belonging to the order Lepidoptera and are classified in three groups according to the pathology caused in their insect hosts. Type 1 pathology is characterized by an infection limited to the host’s midgut and fat body resulting in a relatively slow speed of kill. Type 2 pathology is characterized by infection of most of the host’s tissues and a rapid speed of kill. There is a third pathology with a single representative, the *Harrisina brillians* granulovirus, that causes an infection constrained to the midgut epithelium that results in the rapid death of the host [4].

A highly pathogenic granulovirus was isolated from a larva of the “bean shoot borer” *Epinotia aporema* (Lepidoptera: Tortricidae), one of the major soybean pests in Argentina, and characterized at biological and molecular levels [5]. Further characterization of its pathology demonstrated that this virus belongs to the type 2 GVs meaning that the infection caused by EpapGV in its host is polyorganotropic [6]. All this information has been instrumental to formally propose its use as a microbial control agent with great potential. In order to contribute to a more thorough characterization of EpapGV we set out to determine and analyze its complete genome sequence.

To date, close to 60 baculovirus genomes have been fully sequenced, 12 of them belong to the *Betabaculovirus* genus. Completely sequenced GVs are listed in Table 1 and their pathology types are indicated. In this report, we present the complete sequence and organization of the EpapGV genome and compare them to other baculoviruses using genomic and phylogenetic analyses.

**Table 1:** Completely sequenced Betabaculovirus

**Virus**	**Genome size (bp)**	**Accesion number**	**Annotated ORFs**	**Average % id with EpapGV**	**Host family**	**Pathology type**	**Reference**
EpapGV	119.082	JN408834	133	-	Tortricidae	II	[6], this work.
AdorGV	99.657	AF547984	119	41.02	Tortricidae	I	[7,8]
AgseGV	131.680	AY522332	132	44.12	Noctuidae	II	Xiulian *et al.*, 2004, unpublished
ChocGV	104.710	DQ333351	116	44.39	Tortricidae	nr	[9]
CrleGV	110.907	AY229987	128	44.25	Tortricidae	II	[10]
CpGV	123.500	U53466	143	44.16	Tortricidae	II	[11]
HearGV	169.794	EU255577	179	39.71	Noctuidae	I	[12]
PhopGV	119.217	AF499596	130	42.55	Gelechiidae	II	Croizier *et al.*, 2002, unpublished
PiraGV	108.592	NC_013797	120	44.62	Pieridae	nr	[13]
PlxyGV	100.999	AF270937	120	41.01	Plutellidae	II	[14]
PsunGV	176.677	EU678671	183	40.17	Noctuidae	I	Li *et al.*, 2008, unpublished
SpliGV	124.121	DQ288858	136	40.96	Noctuidae	nr	[15,16]
XcenGV	178.733	AF162221	181	39.84	Noctuidae	I	[17]
ClanGV*	101.487	NC_015398	123	nd	Notodontidae	nr	[18]

When information is available, GV type pathology is indicated. nr: not reported. (*) The genome of *Clostera anachoreta* GV was published [18] after the present work was completed; it was not included in this analysis.

## Results and Discussion

### General characteristics of the EpapGV genome

The complete EpapGV genome [GenBank: JN408834] was covered 34 times by 454 sequencing. It consists of 119,082 bp in good agreement with the previous estimate of 120.1 kbp based on restriction mapping [19]. Betabaculoviruses have AT-rich genomes ranging between 54.7% (CpGV) and 67.6% (CrleGV). The AT content of EpapGV genome is 58.5%. However, no correlation between these data and biological properties has been found thus far. Analysis of the EpapGV genome sequence led to the identification of 133 putative protein coding genes. The search was restricted to open reading frames starting with a methionine codon, coding for polypeptides of at least 50 amino acid residues (aa) and minimal overlapping of adjacent ORFs. This information comprises 90.94% of the nucleotide sequence (Additional File 1). The adenine of the *granulin* start codon was designated nucleotide 1 and the sequence was numbered in the direction of *granulin* gene transcription, which defined the clockwise orientation of the circular genome map [20]. The putative ORFs were numbered sequentially in this orientation. Seventy-two ORFs were in the same orientation as the *granulin* ORF, and sixty-one, in the opposite. EpapGV DNA sequence was searched for promoter motifs 150 bp upstream of the starting codon of each ORF. Early promoter motifs including TATA box (TATAWAW, TATAWTW, TATAW) in conjunction with CAKT initiator sequence (INR) [21] were found in the upstream regions of 26 ORFs; 64 ORFs had a late INR motif DTAAG [22] and 11 ORFs had both early and late elements.

### Gene content

**Figure 1:**
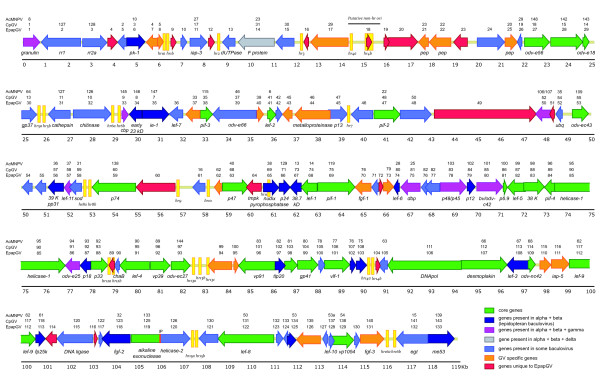
**Linear map of the EpapGV genome.** ORFs and transcription direction are indicated as arrows. ORF number and gene names are indicated above and below arrows, respectively. Homologues in CpGV and AcMNPV are also indicated. Genome position is shown by a kb scale under the thin line. ORF shading is according to key. *Hrs* and putative *non-hr* ori are yellow-shaded. IP: Intervening Polypeptide.

The EpapGV genome contains the 31 core genes present in all baculoviruses. The genes were also classified according to their presence in different genera [23,24] (Figure 1).

**Figure 2:**
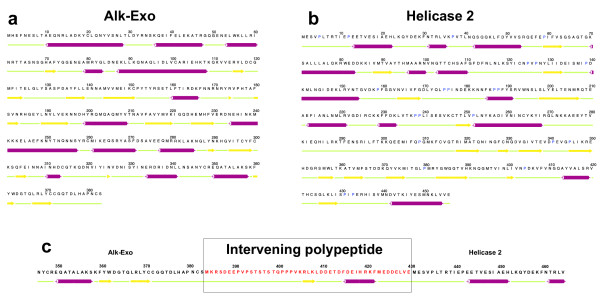
**Predicted secondary structure of a. Alk-Exo, b. Helicase-2 and c. intervening polypeptide.** Prediction was carried out on individual polypeptides and on the fusion protein in three elementary motifs: red cylinders represent α-helix, yellow arrows represent β-sheets, and green lines represent loop regions. Panel **c.** shows the predominance of undefined structure in the intervening polypeptide as well as in the connecting regions between the intervening polypeptide and the C-terminus of Alk-Exo and the N-terminus of helicase-2, respectively.

A distinct feature of the EpapGV genome is that the core gene *alkaline nuclease* (*alk-exo*, *epap119*) is fused in frame with the *helicase-2* ORF (*epap120*). This fusion gene codes for an 886 aa polypeptide with the first 383 residues homologous to Alk-Exo and the last 456 to Helicase-2. A 47 aa-long intervening polypeptide of unknown origin and without significant sequence similarity to any protein in GenBank was found between the Alk-Exo and the Helicase-2 regions. The intervening polypeptide may act as a low-structure linker between Alk-Exo and Helicase-2 such that both enzyme domains could fold as if they were independent polypeptides retaining their respective functions (Figure 2 and Additional files 2 and 3). Although this region was confirmed by resequencing, it will be important to study this genomic region in alternative isolates of EpapGV and determine the transcription and translation products in infected larvae. All GVs contain these two genes in the same order, but there are no reports of a fusion. Fusion genes seem to be extremely rare in baculovirus genomes, but there is one report of fused genes encoded by *Spodoptera litura* NPV: the *ubiquitin* ORF is fused in frame with *gp37* and the fusion protein is proteolytically processed [25].

For two EpapGV ORFs (*epap10* and *epap130*) the BlastP search found homologues in only one member of the *Baculoviridae*. *Epap10* is preceded by early and late promoter motifs and codes for a 90 aa protein that shares 34% amino acid identity with a 88 aa protein encoded by *eppo28* of *Epiphyas postvittana* NPV. This ORF was reported to be unique to EppoNPV and has an early promoter motif [26]. *Epap130* codes for a 77 aa protein that matched a 56 aa protein of *Spodoptera litura* GV (*spli32*) with 38% sequence identity according to ClustalW alignment.

ORFs 10, 46, 54, 55 and 105 were found to have homologues in alphabaculovirus genomes but not in other betabaculoviruses. *Epap46* is a 306 aa long protein that seemed to be homologous to *Spodopera exigua* MNPV ORF 30 by BlastP search (E = 0.07) although they have very low amino acid identity (12%). *Epap54* (148 aa) and *epap55* (157 aa) are both similar to ORF 3 of *Adoxophyes honmai* NPV (AdhoNPV) and ORF 3 of *Adoxophyes orana* NPV (AdorNPV). *Epap55* shares 32% identity with the N-terminal portion of AdorNPV ORF 3 and *Epap54* is homologous to the C-terminal region of ORF 3 of AdorNPV (34%) and AdhoNPV (34%). GV homologues of *epap54* and *epap55* were found only in AgseGV (Additional File 1). *Epap105* is similar to *ac63* of *Autographa californica* MNPV (AcMNPV); their predicted proteins are 28% identical. Its homologue in *Bombyx mori* NPV (BmNPV), *bm51*, was reported to be a structural gene associated with the budded virus (BV) envelope [27], but its deletion resulted in a virus with a phenotype similar to the wild type indicating that it might be a nonessential gene [28].

*Epap24* codes for a 388 aa long protein that is highly similar to ORF 21 of *Cryptophlebia leucotreta* GV (CrleGV) according to BlastP search (E = 4E-05). Crle21 is a 308 aa predicted protein reported to be similar to Se43 [10]. These proteins share a protein motif of the DUF1383 superfamily. They have homologues in all alphabaculoviruses [28] and studies conducted with a deletion mutant of the homologue in AcMNPV (*ac18*) indicated that it is not essential for viral replication both *in vitro* and *in vivo*, but it may play a role in efficient virus infection in *Trichoplusia ni* larvae [29]. Homologues of *epap24*/*crle21* were not found in the rest of the granuloviruses.

### Transcription genes

EpapGV DNA codes for the RNA polymerase subunits *lef-4* (*epap91*), *lef-9* (*epap112*), *lef-8* (*epap122*) and *p47* (*epap63*), *lef-5* (*epap82*) and *vlf-1* (*epap101*), present in all baculoviruses. Additional genes related to the transcription process found in all lepidopteran baculovirus were also detected in the EpapGV genome: *39 k/pp31* (*epap56*), *lef-6* (*epap74*), *lef-11* (*epap57*) (present in gammabaculoviruses) and *pk-1* (*epap6*). *Lef-10*, involved in late transcription and present in most alpha- and betabaculoviruses, was also found in EpapGV genome (*epap128*). Of the baculoviral early transcription genes *ie-0*, *ie-1*, *ie-2* and *pe38*, only *ie-1* (*epap35*) is present in all GVs and pe38 was found in CpGV, CrleGV, PhopGV and PrGV.

### Replication genes

Genes involved in DNA replication that belong to the core group were found in EpapGV genome: *dnapol* (*epap106*), *lef-1* (*epap68*), *lef-2* (*epap41*) and *helicase-1* (*epap85*).

In addition, other genes that belong to this category and were found in EpapGV and in other lepidopteran baculoviruses are *dbp* (*epap75*) (also present in gammabaculoviruses), *lef-3* (*epap108*), *ie-1* (*epap35*), *me53* (*epap133*) and *ac38* (*epap65*). A *lef-7* homologue was found in a BlastP search restricted to baculoviruses: the protein encoded by *epap36* has a match with PsunGV LEF-7 (E = 0.54). This protein was demonstrated to be a baculoviral replication enhancer in AcMNPV [30] and BmNPV [31]. Homologues of this gene are present in group I NPVs, 3 group II NPVs and 3 GVs (XcGV, HearGV [28] and PsunGV)*.*

EpapGV encodes a *DNA ligase* (*epap115*) as do other members of the *Betabaculovirus* genus and three NPVs of group II (LdNPV, LyxyNPV and OrleNPV). This gene seems to be linked to the presence of a second helicase, *helicase-2* (*epap120*) [32], which is fused with alk-exo in EpapGV, but not in the rest of the baculovirus genomes sequenced to date.

### Structural genes

EpapGV genome contains all the structural genes corresponding to the core group as well as the lepidopteran baculovirus genes. The structural core group genes are: *p6.9* (*epap81*), *vp39* (*epap92*), *vp1054* (*epap129*), *vp91* (*epap96*), *gp41* (*epap99*), *odv-ec43* (*epap43*), *odv-e18* (*epap29*), *p74* (*epap59*), *pif-1* (*epap69*), *pif-2* (*epap47*), *pif-3* (*epap38*); *pif-4* (*epap84*); *pif-5/odv-e56* (*epap27*) and the recently discovered *pif-6*[33] (*epap109*). Lepidopteran-specific baculovirus structural genes include *granulin* (*epap1*); *25 k-fp* (*epap113*); *odv-e25* (*epap86*); *bv/odv-c42* (*epap80*), the last two are also present in gammabaculoviruses. *F-protein* (*epap14*) is the only gene shared by alpha-, beta- and deltabaculoviruses. EpapGV contains 42 of the 47 proteins found in the occlusion derived virus (ODV) of PiraGV [34]. Five of these ORFs were only found in betabaculoviruses: *epap48*, *epap94*, *epap95*, *epap123* and *epap126*.

### Auxiliary genes

In addition to core gene *alk-exo* (*Epap119*), some other auxiliary genes were found in EpapGV genome. Viral *ubiquitin* (*epap*52) is present in GVs and all group I alphabaculoviruses. *Cathepsin* (*epap31*) and *chitinase* (*epap32*) were found in some GVs and in most alphabaculoviruses. These genes are responsible for the liquefaction of the host in the final stage of infection [35,36]. Their activity is readily apparent in *E. aporema* larvae infected with EpapGV. There is also a *gp37* (*epap30*) homologue, which is present in some GVs and most NPVs. GP37 is homologous to the entomopoxvirus (EPV) fusolin which was shown to form spindle-like structures. These spindles enhance the peroral EPV infection by contributing to disruption of the peritrophic membrane [37]. EpapGV *gp37* gene has been characterized and demonstrated to be glycosylated [38]. The EpapGV genome includes three fibroblast growth factor homologues: *fgf-1*, *-2* and −*3* (*epap70*, *epap118* and *epap131*, respectively).

The three *fgf* genes are present in all sequenced GVs but *fgf-2* is also present in all alphabaculoviruses. It is thought to be implicated in the virus dissemination in the insect host [39,40]. *Epap58* encodes a superoxide dismutase homologue (*sod*) which is widely distributed in baculovirus. Its potential role is still unknown and controversial [41].

EpapGV also possesses two *iap* genes (inhibitors of apoptosis), *iap-3* (*epap11*) and *iap-5* (*epap111*). *Iap-5* is only present in betabaculovirus whereas *iap-3* is also present in some NPVs. No *p35* homologue was found.

### Granulovirus-specific genes

The number of genes considered to be GV-specific has changed in the literature and will be more accurate when more complete genome sequences become available. These genes could be the basis to the differences between granuloviruses and nucleopolyhedroviruses. Taking into account the analyses presented by Lange *et al*. [10], Wormleaton *et al.*[7], Escasa *et al*. [9], Van Oers & Vlak [42], Miele *et al*. [23] and the present report, a set of 19 genes has been identified in betabaculovirus genomes which were never found in alpha-, gamma- or deltabaculoviruses (Figure 1, Additional file 1). These are EpapGV ORFs 7, 8, 17, 21, 22, 25, 37, 40, 43, 44 (*metalloproteinase*), 62, 70 (*fgf-1*), 73, 94, 95, 110, 111 (*iap-5*), 126 and 131 (*fgf-3*).

Other genes formerly considered as part of the GV-specific set, were dismissed from the list in the present report: CpGV ORFs 30, 32, 45, 50, 56, 77, 82, 119, 121, 122 and 136. All, except cp27, 56, 77, 121 and 136, have homologues in EpapGV but they are absent in some other GV (see Additional file 1).

### Unique genes

Seventeen ORFs appear to be unique to EpapGV compared to the rest of the members of *Baculoviridae* (ORFs 4, 9, 12, 16, 18, 19, 20, 23, 49, 51, 60, 64, 72, 89, 104, 114 and 116). *Epap4* codes for a 144 aa long protein with a conserved motif (COG5152) in its N-terminal region. This motif is an uncharacterized conserved domain that contains RING and CCCH-type Zn-fingers [43]. An early promoter motif was found 150 nt upstream *epap4* ORF. *Epap9* encodes an 81 aa long polypeptide and has no significant BlastP matches. The upstream region contains a GATA motif (TGATAG) and two TATAWAW early promoter elements, but no CAKT INR. *Epap12* codes for a 90 aa protein which shares 23% identity and 43% similarity with a small portion of a 2123 aa protein of *Drosophila ananassae* (XP_001953497); however, no speculation on function can be made. *Epap16* gives no significant BlastP hit, and has early promoter elements upstream of the first ATG (TATAW + 3 CATT elements). Something similar happens with *Epap18* which codes for a hypothetical 76 aa protein and a TATAW element upstream. *Epap19* (94 aa) has no significant BlastP hits and has elements of an early promoter. *Epap20* (422 aa) has no significant BlastP hits and shows elements of a late promoter. *Epap23* codes for a hypothetical protein of 197 aa with no significant similarity with any protein of the GenBank under the control of a putative late promoter and a GATA motif (TGATAG).

*Epap49* codes for the longest hypothetical protein of EpapGV genome (1465 aa). As it lacks characteristic promoter elements and exhibits no similarity with other baculovirus genes it is difficult to predict if it is actually transcribed. *Epap49* is located between the conserved genes *pif-2* (*epap47*, core gene) and *epap50* (homologue to *cp52*). It is worth mentioning that at least in two GVs (HearGV and ChocGV) a similar situation emerged in the same locus. Although a 1144 aa ORF with 27 leucine zippers was initially found in ChocGV, it was not considered a coding sequence but a non-*hr* ori-like region instead; the speculation was based upon its very high AT content (81%), the lack of homology with baculovirus ORFs, and the possibility of sequencing errors (for further details see Escasa *et al*., [9]). In contrast, the 1279 aa ORF in HearGV (*hear44*) was considered a coding sequence resulting from a fusion of the homologues *xc47* and *xc48* of *Xestia c-nigrum* GV [12].

*Epap51* codes for a 69 aa peptide under the control of an early promoter and showed no significant matches in BlastP search. Two GATA motifs (TGATAT and AGATAG) were also found in the region upstream its ATG. *Epap60* (582 aa) shows no significant hits with any protein in the GenBank. TATATAA and TATAA motifs were found upstream the ATG, but without the initiator sequence CAKT, characteristic of early promoters. *Epap64* codes for a thymidylate kinase (described below). *Epap72* codes for a 61 aa peptide with no significant matches in the GenBank under the control of a putative late promoter which is overlapped with a GATA motif (AGATAAG). *Epap89* predicted protein (86 aa) did not have significant BlastP hits either and lacks known promoter motifs except for a TATAAAA sequence 86 nt upstream its ATG overlapped with a GATA motif. Similarly, ORFs 104 (63 aa), 114 (162 aa) and 116 (51 aa) contain TATA box-like motifs upstream the initial ATG and show no significant BlastP hits. *Epap114* also presents a ATAAG sequence, and Epap116 a GATA motif (AGATAA).

### Nucleotide metabolism genes

**Figure 3:**
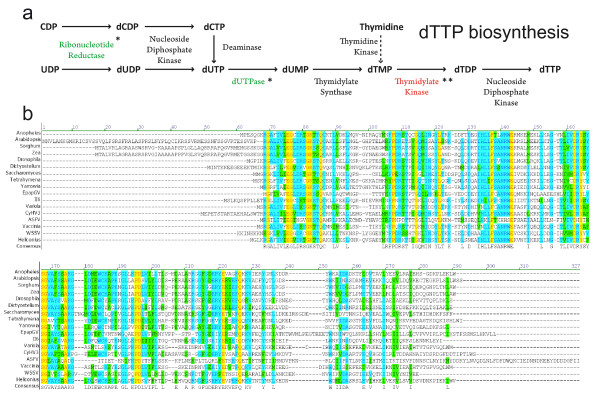
**dTTP biosynthesis and TMPK. a.** Cellular *de novo* pathway of dTTP biosynthesis. Enzymes present in EpapGV and other baculoviruses are marked with an asterisk (*). TMPK gene present in EpapGV and absent in other baculoviruses is highlighted with two asterisks (**). Solid arrows correspond to the *de novo* pathway of dTTP biosynthesis and the dashed arrow to the salvage pathway **b.** Alignment of EpapGV TMPK with other TMPKs from several organisms (only the genus is indicated) and viruses: *Anopheles gambiae* (XP_314179.3); *Arabidopsis thaliana* (NP_001078772.1); *Sorghum bicolor* (XP_002461104.1); *Zea mays* (NP_001150303.1); *Drosophila ananassae* (XP_001960115.1); *Dictyostelium discoideum* (XP_635930.1); *Saccharomyces cerevisiae* (NP_012591.1); *Tetrahymena thermophila* (XP_001009062.1); *Yarrowia lipolytica* (XP_501790.1); II6 (NP_149714.1); Variola Major Virus (NP_042204.1); CyHV3 (YP_001096175.1); ASFV (NP_042729.1); Vaccinia Virus (AAW23610.1); WSSV (AAG40728.1); *Heliconius melpomene* (CBH09285.1). Identical residues conserved in all the sequences are shaded in yellow; those conserved in most sequences are shaded in light blue, and conservative changes are shaded in green.

Genes coding for enzymes involved in nucleotide metabolism have been reported in baculovirus genomes. Ribonucleotide reductase (RNR) catalyses the reduction of ribose in ribonucleotide diphosphates to yield deoxyribonucleotides, the building blocks of DNA [44]. In most eukaryotes the active RNR is a tetrameric complex made up of homodimers of two subunits coded by genes *rr1* and *rr2*, which have been also found in some NPVs and 4 GVs: CpGV, AgseGV, PhopGV and EpapGV (*epap2* and *epap3*). On the other hand, dUTPase catalyses the dephosphorylation of dUTP to yield dUMP. As dUTP can be mutagenic if incorporated in DNA, this enzyme helps to keep levels of dUTP low and prevents its incorporation in DNA, in lieu of dTTP. This gene is present in some NPVs and in the betabaculoviruses AgseGV, SpltGV and EpapGV (*Epap13*). The presence of *rr1*, *rr2* and *dutpase* appears to be linked in the genomes of OpMNPV, SpltNPV, SeMNPV and LdMNPV [32]. This linkage appears in the betabaculoviruses AgseGV and EpapGV but not in SpltGV. Both enzymes participate in the pathway of *de novo* dTTP biosynthesis (Figure 3a).

EpapGV codes for a novel enzyme in the family *Baculoviridae*, which also takes part in this pathway: *epap64* codes for a predicted 224 aa protein homologous to thymidylate kinase, also known as thymidine monophosphate kinase (TMPK), that catalyses the phosphorylation of dTMP to produce dTDP. BlastP hits included different eukaryotic organisms and several viruses representing the families *Poxviridae* (Variola Virus), *Iridoviridae* (Invertebrate iridescent virus 6, II6), *Herpesviridae* (Cyprinid Herpes 3, CyHV3), *Nimaviridae* (White Spot Syndrome Virus, WSSV) and *Asfaviridae* (African swine fever virus, ASFV) that were used in the ClustalW alignment with EpapGV TMPK (Figure 3b). EpapGV TMPK showed the highest identity (40%) with TMPK from the insect *Drosophila ananassae* and the least (22%) with TMPK from ASFV. The degree of identity with the other viruses was 35% (II6); 32% (Variola and Vaccinia); and 33% (WSSV). Besides *Baculoviridae*, other viral families that encode nucleotide metabolism genes include *Herpesviridae*, *Poxviridae* and *Asfaviridae*. The alphaherpesvirus pyrimidine deoxynucleoside kinase, popularly known as thymidine kinase (TK) phosphorylates a wide range of nucleoside substrates, as well as TMP (TK + TMPK activity)**,** and is responsible for the rise in the TTP pool characteristic of HSV-infected cells [45]. In poxviruses these TK and TMPK activities are present in separate enzymes as happens in cellular organisms. Vaccinia virus TMPK was found to be nonessential for virus replication in cultured cells and able to complement a *tmpk- Saccharomyces cerevisiae* mutant [46].

The White Spot Syndrome Virus (WSSV; *Nimaviridae*) genome contains a mosaic gene that encodes a *tk-tmpk* fusion of both homologues, *i.e.* cellular-type thymidine kinase TK1 and cellular-type TMPK [47]. However, only TK activity, but not TMPK, could be demonstrated for WSSV TK-TMK [48]. TMPK substrate specificity was studied in vaccinia virus and it was found to phosphorylate dTMP, dUMP and, unlike human TMPK, dGMP as well [49]. EpapGV TMPK expression and substrate specificity, as well as its role in infection, remain to be elucidated.

### Repeated sequences

A common feature in baculovirus genomes is the presence of nucleotide sequence repeats known as homologous regions (*hrs*). These regions function as enhancers of early gene transcription and are thought to play a role as origins of replication. They are characterized by tandem copies of sequence motifs that include an imperfect palindromic core. Although they present significant sequence similarity within a genome they are highly variable when compared between any two different species (Reviewed in [50]).

**Figure 4:**
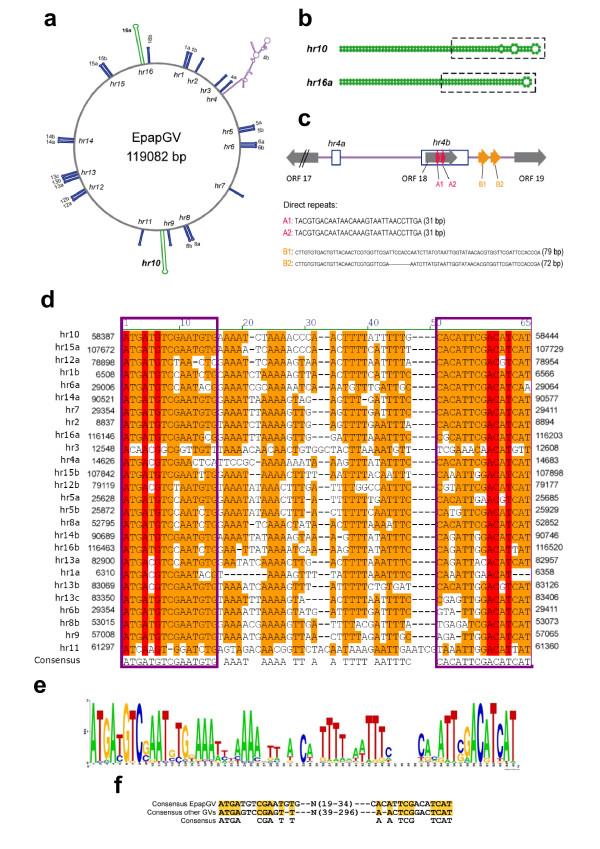
**Repetitive sequence analysis. a.** Distribution of the putative *hrs* in the EpapGV genome with their respective palindromes. **b**. Predicted secondary structure of the two largest palindromes. The central palindrome used in the multiple alignment is boxed with a dashed line. **c.** Structure of the putative *non-hr* ori. **d.** Alignment of all the palindromes excluding *hr4b*. Genome positions are indicated at both ends of the aligned sequences. Conserved 15 bp ends are boxed. **e.** Sequence logo performed with the multiple alignment of the palindromes. **f.** Comparison between consensus EpapGV palindromic ends with the consensus 13 bp ends reported by Hilton & Winstanley (2008) [51] (see Figure 3 of this reference).

In a first screening of the EpapGV genome for repeated sequences with Blast2seq we found two palindromic regions, of 128 bp (58352–58479) and 122 bp (116114–116235), respectively. Both sequences are very likely to exist in equilibrium between double stranded DNA and opposite hairpin-loops constituted by each complementary strand (*hr*10, -75.30 kcal/mol and *hr*16a, -86.50 kcal/mol, respectively) forming a cruciform-like structure (Figure 4a,b). Whether this feature is biologically relevant remains to be elucidated. Using these two sequences as profiles we searched the rest of the genome for similar sequences. Twenty-four AT-rich sequences of similar size were detected dispersed throughout the genome. The alignment of these sequences revealed the presence of conserved palindromes of about 58 bp that correspond with the central part of the two initially identified largest palindromes, with a mean of 70% AT content. The alignment of these shorter palindromes (Figure 4d) shows that they have an AT rich core flanked by 15 bp conserved inverted repeats (Figure 4d,e). This structure is similar to that of *hrs* found in all GVs (those sequenced to date) that infect other insects of the Tortricidae family (CpGV, CrleGV, AdorGV, ChocGV): palindromic sequences of about 63–76 bp characterized by conserved blocks of 13 bp located at both ends. These ends were found to be similar in sequence not only among the palindromes of a single genome but among the different genomes, as well [51]. EpapGV palindromic ends are similar to the consensus 13 bp sequence for these GVs, including PhopGV (Figure 4f). PhopGV, that infects a member of the Gelechiidae family but appears in the same clade as these GVs, was also found to have this kind of palindromic repeats, although their length was different (142–320 bp) [51]. Palindromes sharing these features were analyzed in infection-dependent replication assays in cells susceptible to CpGV infection. All 14 CpGV palindromes were found to act as origins of replication of plasmids in infection-dependent assays in *Cydia pommonela* cells [51,52], whereas the *hrs* from other GVs tested in the same way did not show this ability, with the only exception of 2 palindromes of the most closely related CpGV virus, CrleGV [51].

These palindromic sequences are found with a much greater frequency in EpapGV DNA compared to the other tortricid-specific GVs. EpapGV contains 26 palindromes (within 16 *hrs*), whereas the others have up to 17 palindromes as is the case of CrleGV [51]. In contrast, only four *hrs* (each one containing only 2–3 direct repeats) were reported in the most recently published betabaculovirus genome sequence (ClanGV; [18]).

There seem to be some conserved locations for the *hrs* in GV genomes. For example, the region between *sod* and *p74* and downstream of the CpGV ORF 5 [51].

It has been reported for AcMNPV that VLF-1 (a protein present in all the baculoviruses sequenced to date) binds with high affinity to cruciform DNA structures and it was suggested that this may play an important role in the replication/packaging process [53]. These cruciform structures, formed by the two largest palindromes or by the smaller ones interspersed in the EpapGV genome, may as well interact with VLF-1 and play a role in the replication or packaging.

In addition to the 26 palindromes mentioned above, there is a large structure consisting of 327 bp flanked by the 15 bp conserved ends predicted to form the secondary structures shown in Figure 4(a). This structure is located in the *hr4* region (including *hr4a* and *hr4b*), an AT-rich sequence between ORFs 17 and 19. The sequence organization is depicted in Figure 4(c) showing ORF 18 within *hr4b*, which also contains two 31 bp direct repeats (A1, A2), and an intergenic region with a second pair of imperfect direct repeats of 79 and 72 bp (B1, B2), respectively. Interestingly, this region is located in the same relative position where a putative non-*hr* ori was described in CpGV spanning ORFs 24, 25 and 26 (which are absent in EpapGV) [11] and in CrleGV [54].

### Relationships with other baculoviruses

**Figure 5:**
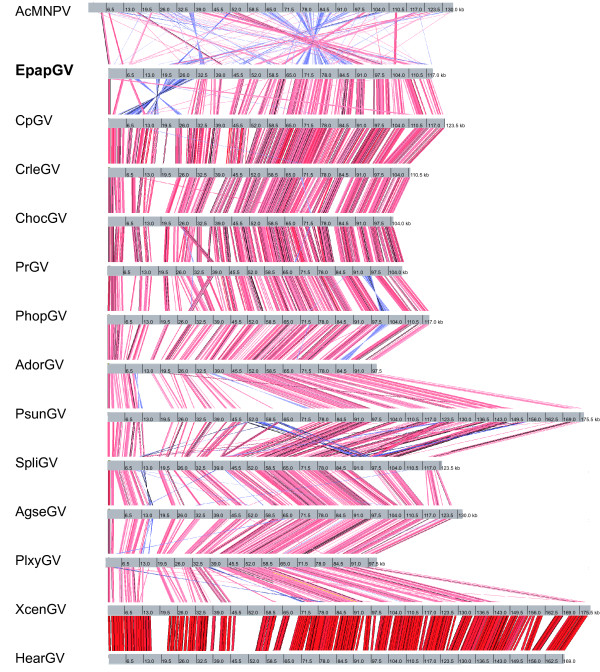
**Syntenic map of EpapGV and other baculovirus genomes.** Comparison of gene colinearity among 13 GVs and AcMNPV. Each genome is represented by a grey line where nucleotide positions are indicated (kb). Red stripes connecting the genomes indicate syntenic regions in the same strand, whereas blue stripes indicate syntenic regions in opposite strands (inversions). Color intensity is proportional to % identity (darker is more conserved).

Strong colinearity is observed in granulovirus genomes sequenced to date [7,10,11,51]. Baculovirus gene colinearity has been analysed mainly by Gene Parity Plot [55]. In this study we used the Artemis Comparison Tool to analyse the gene synteny of EpapGV compared to all other sequenced GVs and the type species of the *Alphabaculovirus* genus, AcMNPV. This tool enables to construct synteny maps through a tBlastX comparison between genomes, where inversions are easily detected as well as the different percentages of identities that correlate with different colour intensity. Figure 5 shows the conserved gene colinearity of all 13 sequenced GV genomes and the poorly conserved synteny between GVs and AcMNPV. Notably EpapGV differs from the rest of the GVs by a *ca.* 20 kb gene block inversion, as we noted previously in a physical map [19].

**Figure 6:**
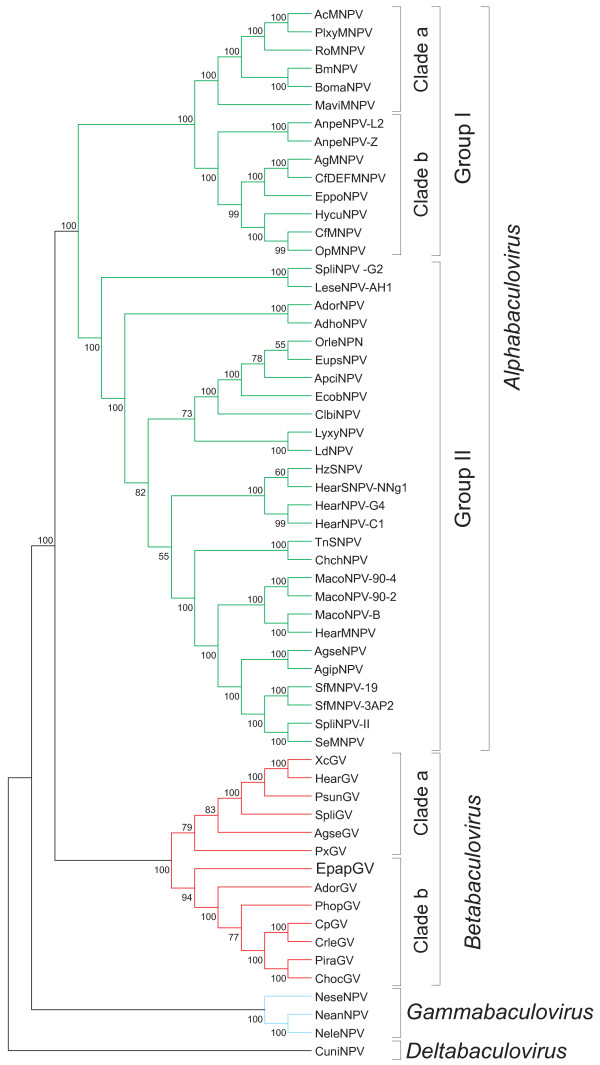
**Phylogenetic tree.** Cladogram based on amino acid sequences of 31 core genes obtained from 58 available complete baculoviral genomes were individually aligned, concatenated and a phylogenetic tree was inferred with MEGA 5 program. The four Baculovirus genera and the different subgroups are indicated.

Phylogenetic analysis based on 31 concatenated core genes of 58 baculovirus genomes was performed (Figure 6). The obtained cladogram reproduced the grouping of four genera recognized in the current classification of the family [1]. Division in two main groups of the *Alphabaculovirus* genus agrees with the group I and II. Two clades (Ia and Ib) previously described in group I by using concatenated amino acid sequences of the partial *polh/gran*, *lef-8* and *lef-9* genes [56] were also confirmed in our analysis.

As expected, EpapGV grouped in the *Betabaculovirus* genus. In previous reports it was observed that totricidae and noctuidae specific GVs tend to be in separated groups [56,57]. The cladogram obtained in this work confirms previous observations, and the additional complete genomes considered here allowed the division of betabaculoviruses in two well separated monophyletic clades as reported previously [23]. Clade “a” includes six species: PxGV, AgseGV, SpliGV, PsunGV and XcGV, which were isolated mainly from *Noctuidae* hosts. PxGV is the exception; its host belongs to the *Plutellidae* family. Clade “b” includes seven species: EpapGV, AdorGV, PhopGV, CpGV, CrleGV, PiraGV and ChocGV; five of them were isolated from *Tortricidae*, whereas PiraGV was isolated from *Pieridae* and PhopGV, from *Gelechiidae*. EpapGV seems to be the GV isolate closest to the common ancestor of Clade “b”. Both clades includes slow killing (type 1 GVs) and fast killing (type 2 GVs), reinforcing the concept of that this biological feature is not phylogenetically informative [57].

## Conclusions

In this study the complete genome of EpapGV was characterized. It includes genes that are common to all baculovirus, and others that have been found only in some of the isolates; in addition, it contains 17 genes that are not shared with the rest of the family: 16 with unknown functions, and one encoding a TMPK homologue which may have been captured from of a host genome or a different coinfecting pathogen. Also a set of 19 betabaculovirus-specific genes, was determined. The information collected and analyzed in this study provides ground for further investigations to improve the understanding of the molecular steps involved in EpapGV infection.

The analyses of gene order and identity suggest that evolution of baculoviruses occurred via acquisition of both individual genes (or gene fragments) and larger blocks of host DNA sequences followed by events of inversions, deletions, and re-acquisitions of previously lost sequences. Interestingly, the phylogenetic analysis suggested that EpapGV is situated closest to the common ancestor of clade b *Betabaculovirus*.

## Methods

### Insects, virus and viral DNA

EpapGV was originally isolated from a larva of the bean shoot borer *Epinotia aporema* collected in Oliveros (Santa Fe, Argentina) [5]. It was amplified allowing fourth instars to feed on artificial diet superficially contaminated with EpapGV occlusion bodies (OBs). Moribund larvae were collected and processed according to Parola *et al.*[19]: viral DNA was isolated from sucrose gradient purified OBs. Its integrity and identity was checked by restriction digestion and agarose gel electrophoresis.

### Nucleotide sequence determination and analysis

EpapGV genomic DNA was sequenced with the 454 Genome Sequencer (GS) FLX™ Standard (Roche) at the Interdisciplinary Center for Biotechnology Research (ICBR), University of Florida (Gainesville, US). *De novo* assembly was generated on newBler assembler (GS FLX Data Analysis Software).

Open reading frames (ORFs) were identified using VectorNTI software (Invitrogen) and ORF Finder http://www.ncbi.nlm.nih.gov/gorf/gorf.html[58]. ATG initiated ORFs of at least 150 nt (50 aa) with minimal overlap were selected for further analysis. Homology searches were done using Blast [59]. Percentage identities between homologous genes were obtained by global alignments with ClustalW [60] using default parameters. Early (E) and late (L) Promoter motifs within 150 bp upstream of the putative ORFs were screened. E indicates the presence of a TATA-box (TATAW, TATAWAW, TATAWTW) with a CAKT mRNA start site 20–40 nucleotides downstream; whereas L denotes a DTAAG sequence [7,10,61]. Also GATA motifs WGATAR [62] and WGATAY [63] were searched for the unique genes.

Prediction of secondary structure of Alk-Exo_Helicase-2 fused protein was performed with the Jpred3 server [64]; http://www.compbio.dundee.ac.uk/www-jpred/) using default parameters and single sequence submit. Actually, the prediction accuracy of Jnet (main Jpred3 algorithm) raised 81.5% in blind tests with soluble proteins. C-terminal end of Alk-Exo and N-terminus of Helicase-2 were selected on the basis of multiple alignments of the respective GV proteins.

Repeated sequences were searched first aligning EpapGV genome to itself through Blast2seq program from NCBI [65]. The first hit which corresponds to the 100% match of the complete genome was ignored and the following hits were used for further analysis. The consensus alignment obtained from two palindromes was used to find similiar sequences along the genome with the VectorNTI program (Invitrogen). The secondary DNA structure prediction of these sequences were performed in the Mfold server of The Vienna RNA website [66]. The alignment of all the palindromes found was performed with ClustalW algorithm with default parameters. The sequence logo of this alignment was carried out at the WebLogo server (http://weblogo.berkeley.edu/) [67].

EpapGV genome was compared with other baculovirus genomes by constructing syntenic maps with the Artemis Comparison Tool (ACT) [68] (The Sanger Institute; http://www.sanger.ac.uk/resources/software/act/), using tBlastX program.

Phylogenetic analysis was performed using 31 core genes from 58 baculovirus genomes (Additional File 4) which were independently aligned using ClustalX program [69], with the following parameters: Pairwise alignment (Gap Open Penalty = 10, Gap Extension Penalty = 0.1, protein weight matrix: Blosum 30); Multiple alignment (Gap Open Penalty = 10, Gap Extension Penalty = 0.05, protein weight matrix: Blosum series). Then a concatemer was generated by addition of the complete individual alignments and phylogeny was inferred using MEGA 5 program [70] with the following parameters: UPGMA; Bootstrap with 1000 replicates; gap/Missing data = complete deletion; Model = Amino (Dayhoff Matrix); patterns among sites = Same (Homogeneous); rates among sites = Different (Gamma Distributed); gamma parameter = 2.25. The obtained data was deposited in TreeBASE (http://purl.org/phylo/treebase/phylows /study/TB2:S12862).

## Competing interests

The authors declare that they have no competing interests.

## Authors' contributions

MLF obtained and purified the viral DNA, analyzed the sequence and wrote the manuscript. RS and MEB participated in viral amplification and purification from insect larvae and revised the manuscript. RS also assisted with the phylogenetic analysis. SH participated in the sequence analysis and revised the manuscript. MFB participated in the hrs analysis and discussion and revised the manuscript. ASC is one of the senior scientists in the project, she provided the viral isolate and equipment for virus and DNA purification, advised on the study and revised the manuscript. PDG participated in the sequence analysis, the phylogenetic tree and supplementary figures, advised on the study and revised the manuscript. VR conceived the study and revised the manuscript. All authors approved the final manuscript.

## Authors' information

MLF and HS hold fellowships from Consejo Nacional de Investigaciones Científicas y Técnicas (CONICET); RS an MEB held fellowships from ANPCyT and CONICET, respectively, when they participated in this study. VR, PDG and MFB hold research career awards from CONICET; ASC and MFB are staff researchers at IMYZA (CICVyA, INTA).

## Supplementary Material

Additional file 1 **Predicted ORFs in the genome of EpapGV.** This file lists the ORFs predicted in the genome of EpapGV and their homologues in other completely sequenced betabaculoviruses. Click here for file

Additional file 2 **Multiple alignment of betabaculovirus alkaline exonuclease.** This file shows the alignment of betabaculovirus alkaline exonuclease amino acid sequences. Click here for file

Additional file 3 **Multiple alignment of betabaculovirus helicase 2.** This file shows the alignment of betabaculovirus helicase 2 amino acid sequences. Click here for file

Additional file 4 **Characteristics of baculovirus genomes.** This file lists the characteristics of baculovirus genomes, including virus name, genome size, number of ORFs and GeneBank accession number. Click here for file
