# Supplementary material for: Genome of Epinotia aporema granulovirus (EpapGV), a polyorganotropic fast killing betabaculovirus with a novel thymidylate kinase gene
Source: BMC Genomics. 2012 Oct 11;13:548. doi: 10.1186/1471-2164-13-548 (PMC3496565; doi:10.1186/1471-2164-13-548)
Supplement: Additional file 1 — Predicted ORFs in the genome of EpapGV. This file lists the ORFs predicted in the genome of EpapGV and their homologues in other completely sequenced betabaculoviruses. [file 1471-2164-13-548-S1.pdf]

EpapGV ORFs

ORF	Name	Location	Length (aa)	Prom. Motifs	Homologues (% identity) Polypeptide Length<br>CpGV	CrleGV	AdorGV	PhopGV	PlxyGV	XcenGV	AgseGV	HearGV	ChocGV	SpltGV	PsunGV	PiraGV	AcMNPV
1	*Granulin*	1 > 747	249	E, L	1 (92) 248	1 (93) 248	1 (93) 248	1 (90) 248	1 (86) 248	1 (88) 248	1 (87) 248	1 (88) 248	1 (91) 248	1 (89) 249	1 (88) 194	1 (83) 247	8 (55) 243
2	*rr1*	803 < 2584	593	E	127 (50) 624			119 (53) 609			37 (64) 615						
3	*rr2a*	2672 > 3772	366	L	128 (52) 362			120 (55) 345			38 (60) 369						
4		3809 > 4246	145	E													
5		4256 < 4669	137	E	2 (21) 174	2 (28) 164	2 (28) 106	2 (24) 163	5 (15) 131	2 (19) 231	2 (29) 222	2 (19) 233	2 (25) 126	2 (25) 189	2 (15) 240	2 (27) 137	
6	*pk-1*	4668 > 5498	276		3 (41) 279	3 (48) 310	3 (48) 275	3 (49) 278	6 (50) 274	3 (52) 302	3 (50) 384	3 (52) 299	3 (44) 277	3 (50) 282	3 (51) 284	3 (45) 278	10 (33) 272
7		5493 < 6074	194	L	4 (35) 188	4 (36) 188	4 (28) 191	4 (38) 190	8 (24) 175	7 (31) 187	6 (37) 184	6 (31) 187	5 (33) 187	5 (34) 189	6 (31) 187	4 (37) 188	
8		6055 > 6327	90		5 (15) 80	5 (13) 86	5 (12) 83	5 (26) 91	9 (28) 93	8 (13) 86	7 (23) 87	7 (12) 86	6 (12) 84	6 (22) 77	7 (12) 86	5 (18) 79	
9		6660 > 6905	81														
10		7110 > 7382	90	E, L													
11	*iap-3*	7522 > 8292	256		17 (60) 275	16 (52) 255	89 (42) 254	108 (31) 271	98 (26) 281	137 (25) 285	53 (42) 269	138 (25) 284	84 (57) 283	16 (50) 97	143 (26) 290	79 (32) 217	27 (29) 286
12		8364 > 8636	90														
13	*dUTPase*	8991 < 9563	190								17 (44) 192			51 (58) 140			
14	*F protein*	9703 < 11328	541		31(48) 601	30 (49) 592	23 (41) 574	27 (47) 595	26 (42) 544	27 (35) 599	25 (51) 598	26 (35) 581	23 (50) 586	24 (41) 585	26 (36) 581	26 (50) 589	23 (16) 690
15		11401 < 12228	275		30 (19) 181			26 (19) 179	25 (16) 431	26 (15) 370	24 (17) 362	25 (16) 370		23 (14) 217			
16		12635 > 12850	71														
17		12923 < 14527	534		29 (17) 304	28 (14) 286	21 (15) 373	25 (8) 380	24 (13) 388	25 (11) 449	23 (14) 451	24 (11) 469	22 (13) 416	22 (11) 410	24 (11) 437	24 (12) 429	
18		15301 > 15531	76														
19		15974 > 16228	94	E													
20		16218 > 17486	422	L													
21	*pep*	17518 > 17946	142		23 (52) 152	24 (51) 159	18 (48) 128	21 (56) 146	23 (50) 131	18 (49) 153	20 (55) 146	17 (48) 153	19 (59) 134	18 (47) 144	17 (48) 153	22 (50) 145	
22	*pep/p10?*	17976 < 18902	308	L	22 (60) 347	23 (62) 326	17 (58) 336	20 (55) 302	21 (53) 320	19 (52) 386	19 (60) 350	18 (52) 386	18 (61) 330	19 (44) 370	18 (52) 387	21 (60) 340	
23		19013 < 19606	197	L													
24		20022 > 21188	388			21 (22) 309											18 (10) 353
25	*pep*	21206 > 21739	177		20 (60) 235	20 (63) 207	16 (50) 203	19 (58) 157	20 (46) 235	17 (54) 187	18 (59) 206	16 (52) 187	17 (64) 163	15 (53) 215	16 (54) 190	20 (52) 204	
26		21735 < 21932	66		19 (21) 74	19 (28) 64	15 (19) 60		17 (36) 58	16 (47) 71	16 (36) 74	15 (45) 71	16 (28) 56		15 (43) 68	19 (20) 78	29 (16) 71
27	*odv-e56*	22035 > 23099	354	L	18 (70) 355	17 (69) 353	12 (67) 359	16 (68) 351	16 (64) 351	15 (52) 353	15 (55) 352	14 (53) 353	14 (72) 353	13 (52) 365	14 (53) 353	16 (68) 354	148 (42) 376
28		23152 > 24525	457	E, L	15 (43) 457	14 (45) 451	11 (43) 459	13 (36) 426	14 (41) 446	13 (40) 453	12 (44) 446	12 (40) 453	13 (43) 455	11 (38) 456	12 (40) 452	15 (43) 460	142 (29) 477
29	*odv-e18*	24526 > 24792	88	E, L	14 (76) 84	13 (80) 85	10 (80) 87	12 (80) 83	13 (49) 91	12 (67) 83	11 (65) 92	11 (69) 83	12 (84) 88	10 (68) 80	11 (70) 83	14 (79) 66	143 (40) 62
30	*gp37*	24830 > 25498	222	L	13 (50) 251					107 (42) 244		109 (42) 244			110 (45) 248	13 (49) 240	64 (48) 302
31	*cathepsin*	26119 < 27108	329	L	11 (57) 333	11 (57) 332				58 (48) 346	31 (59) 327				65 (51) 338	11 (57) 339	127 (44) 323
32	*chitinase*	27245 > 28957	570	L	10 (61) 594					103 (59) 594	32 (61) 585	105 (60) 585			108 (60) 585	10 (62) 586	126 (60) 551
33	*chitin binding*	29439 < 29735	98	L	9 (59) 101	8 (62) 123	8 (50) 102	8 (50) 123	12 (64) 101	11 (52) 99	10 (53) 99	10 (52) 99	9 (62) 98	9 (53) 99	10 (50) 99	8 (64) 101	145 (29) 77
34	*early 23 kD*	29774 < 30349	191		8 (29) 192	7 (32) 189	7 (27) 191	7 (27) 205	11 (18) 180	10 (24) 196	9 (32) 200	9 (24) 196	8 (27) 254	8 (26) 205	9 (26) 195	7 (30)186	146 (17) 201
35	*ie-1*	30368 > 31564	398		7 (34) 488	6 (32) 454	6 (32) 422	6 (32) 426	10 (29) 393	9 (32) 484	8 (32) 463	8 (32) 485	7 (31) 446	7 (29) 432	8 (31) 474	6 (34) 460	147 (16) 582
36	*lef-7*	31575 < 32210	212							129 (16) 312		132 (16) 312			134 (19) 322		
37		32332 < 32967	211	L	33 (30) 294	32 (33) 234	25 (30) 284	29 (27) 220	28 (37) 210	29 (27) 170	27 (36) 274	28 (30) 240	24 (32) 239	26 (34) 229	28 (29) 236	28 (32) 316	
38	*pif-3*	32979 > 33536	185	L	35 (53) 199	34 (48) 185	26 (37) 187	31 (40) 177	29 (48) 181	32 (44) 195	29 (48) 191	30 (44) 195	26 (42) 194	27 (46) 215	30 (44) 194	30 (46) 190	115 (33) 204
39	*odv-e66*	33544 < 35508	654	L	37 (47) 683	35 (45) 697	28 (47) 747	33 (47) 796	30 (48) 682	149 (49) 668	33 (49) 678	150 (31) 668	27 (19) 729	125 (38) 715	156 (38) 667	44 (37) 762	46 (45) 704
40		35532 > 35840	102	L	39 (56) 105	36 (54)105	29 (50) 104	34 (55) 104	31 (38) 129	34 (36) 113	34 (40) 111	32 (36)113	28 (55) 103	31 (33) 116	32 (36) 113	31 (53) 104	
41	*lef-2*	36020 > 36403	127		41 (43) 171	38 (45) 168	32 (38) 170	37 (45) 180	32 (41) 270	35 (39) 189	35 (46) 181	33 (39) 189	29 (49) 188	32 (41) 174	33 (42) 194	33 (45) 172	6 (23) 210
42		36397> 36681	94		42 (26) 82	39 (23) 82	33 (20) 87	38 (29) 85		36 (23) 89	36 (27) 83		30 (26) 83	33 (26) 81	34 (22) 89	34 (29) 82	
43		36698 < 37132	144		45 (19) 154	42 (11) 98	36 (24) 153	40 (16) 117	34 (13) 133	39 (16) 149	40 (19) 142	37 (17) 149	32 (20) 150	36 (12) 140	37 (12) 152	36 (19) 150	
44	*mp-nase*	37203 < 38768	521	L	46 (30) 545	43 (31) 486	37 (30) 395	41 (29) 469	35 (29) 402	40 (29) 469	41 (33) 481	38 (27) 596	33 (30) 488	37 (28) 464	38 (26) 593	37 (35) 432	
45	*p13*	38689 > 39519	276	L	47 (58) 269	44 (56) 270	38 (54) 266	42 (55) 277	36 (50) 263	43 (51) 277	42 (56) 265	40 (51) 277	34 (61) 268	38 (52) 267	41 (51)278	38 (53) 266	
46		39687 < 40607	306	E, L													

47	*pif-2*	40662 > 41786	374	L	48 (64) 372	45 (62) 373	39 (56) 375	44 (56) 366	37 (58) 368	45 (48) 388	43 (53) 382	42 (49) 388	35 (65) 374	39 (54) 378	43 (49) 388	40 (61) 375	22 (49) 382
48		41789 > 43129	446	L	50 (10) 727	47 (14) 714	42 (14) 875	46 (11) 675	38 (21) 153	47 (18) 220	45 (15) 840	44 (13) 1279		41 (11) 848	45 (13) 1213	42 (12) 803	
49		43254 > 47651	1465														
50		47649 < 48287	213	L	52 (55) 342	50 (61) 206	43 (54) 202	47 (58) 302	40 (54) 206	50 (51) 272	46 (57) 219	45 (50) 257	37 (57) 210	42 (51) 220	47 (49) 260	43 (61) 210	106 (33) 61
51		48253 < 48459	69	E													
52	*ubiquitin*	48485 < 48766	93	L	54 (83) 94	52 (86) 76	45 (73) 94	49 (78) 94	42 (80) 114	52 (82) 97	47 (70) 97	47 (82) 77	39 (80) 95	45 (78) 93	50 (82) 77	45 (75) 95	35 (77) 77
53	*odv-ec43*	48844 > 49887	348	L	55 (48) 326	53 (54) 348	46 (53) 349	50 (46) 269	43 (47) 414	53 (43) 353	48 (61) 373	48 (43) 353	40 (57) 343	46 (47) 358	51 (44) 353	46 (57) 354	109 (32) 390
54		50019 < 50465	148								89 (26) 435						
55		50602 < 51075	157	E, L							90 (23) 435						
56	*39k/pp31*	51176 < 51916	247	E	57 (25) 241	55 (28) 255	49 (30) 278	52 (37) 231	45 (27) 252	55 (24) 295	51 (35) 274	50 (25) 293	42 (27) 251	48 (25) 285	53 (24) 298	48 (30) 287	36 (11) 275
57	*lef-11*	51897 < 52211	104		58 (40) 134	56 (45) 94	50 (44) 94	53 (44) 88	46 (49) 96	56 (48) 102	52 (46) 106	51 (48) 102	43 (42) 92	49 (46) 98	54 (49) 95	49 (44) 92	37 (22) 112
58	*sod*	52132 < 52683	183	L	59 (65) 132	57 (65) 151	51 (58) 152	54 (61) 166	47 (61) 153	68 (68) 153	54 (65) 156	63 (67) 154	44 (61) 161		64 (66) 153	50 (67) 156	31 (58) 151
59	*p74*	53154 < 55115	653	L	60 (55) 688	58 (53) 670	53 (50) 642	55 (51) 658	49 (49) 578	77 (44) 710	56 (47) 675	72 (43) 710	46 (53) 664	56 (30) 144	77 (42) 710	51 (55) 677	138 (40) 645
60		55140 < 56888	582														
61		57682 < 58236	184	E	16 (42) 196	15 (47) 197		14 (42) 205			14 (48) 211						
62		58654 < 58974	106	L	62 (37) 189	60 (40) 104	57 (17) 66	60 (34) 172	50 (35) 135	77a	59 (31) 155	73 (34) 104	49 (37) 120	57 (38) 87	78 (30) 111	55 (42) 147	
63	*p47*	59013 < 60200	395		68 (59) 460	61 (59) 387	58 (53) 388	61 (59) 399	51 (58) 386	78 (56) 394	60 (57) 403	74 (56) 394	50 (60) 385	58 (56) 397	79 (56) 394	56 (63) 399	40 (42) 401
64	*tmpk*	60092 < 60766	224														
65	*Nudix hidrolase*	60861 < 61538	226		69 (57) 220	62 (58) 221	59 (52) 225	62 (51) 215	52 (58) 207	79 (62) 225	61 (64) 220	77 (62) 225	51 (54) 217	59 (61) 222	82 (63) 225	57 (58) 221	38 (36) 216
66	*p24*	61570 > 62067	165	L	71 (55) 203	63 (56) 183	60 (52) 156	63 (47) 167	53 (59) 159	80 (48) 182	62 (61) 177	78 (48) 182	52 (56) 182	60 (53) 162	83 (46) 171	58 (51) 217	129 (24) 198
67	*38.7 kD*	62086 < 62571	161		73 (22) 198	65 (27) 151	61 (16) 188	65 (25) 185	54 (29) 150	81 (22) 184	63 (29) 193	79 (22) 184	54 (32) 166	61 (18) 153	84 (24) 192	59 (28) 174	13 (18) 327
68	*lef-1*	62555 < 63256	233		74 (56) 235	66 (54) 235	62 (55) 233	66 (57) 233	55 (53) 251	82 (48) 238	64 (52) 253	80 (48) 238	55 (58) 233	62 (53) 277	85 (46) 238	60 (57) 233	14 (34) 266
69	*pif-1*	63280 > 64896	538	L	75 (56) 538	67 (54) 535	63 (51) 533	67 (47) 522	7 (53) 536	84 (46) 540	65 (35) 547	82 (47) 540	56 (57) 529	64 (47) 538	87 (46) 541	61 (54) 541	119 (35) 530
70	*fgf-1*	64896 < 65570	225		76 (27) 195	68 (27) 230	64 (30) 231	69 (32) 239	56 (28) 221	85 (20) 232	66 (30) 225	83 (20) 232	57 (32) 223	66 (30) 232	88 (21) 233	62 (30) 226	
71		65613 < 65927	104	E				70 (29) 105					58 (22) 100			63 (25) 99	
72		65935 > 66120	61	L													
73		66113 > 66571	152	E, L	79 (29) 156	70 (28) 154	66 (27) 151	71 (29) 152	59 (22) 79	87 (30) 164	67 (25) 108	85 (30) 164	59 (27) 152	68 (30) 81	90 (28) 164	64 (26) 160	
74	*lef-6*	55564 < 66812	82		80 (33) 101	71 (30) 102	67 (26) 102	72 (27) 94	60 (29) 86	88 (30) 99	68 (34) 82	86 (32) 99	60 (31) 84	69 (32) 99	91 (33) 99	65 (26) 104	28 (15) 173
75	*dbp*	66873 < 67712	279		81 (26) 290	72 (25) 284	68 (27) 336	73 (28) 277	61 (25) 263	89 (18) 277	69 (33) 296	87 (18) 277	61 (28) 275	70 (23) 270	92 (19) 277	66 (28) 281	25 (14) 316
76		67749 < 67952	67		82 (23) 306	73 (20) 284			62 (23) 136		70 (29) 100	88 (20) 70	62 (21) 86	71 (31) 50	93 (18) 70		
77		67906 < 68580	224		82 (19) 306	73 (25) 284		74 (13) 175		90 (13) 246	71 (23) 292	89 (13) 246	63 (22) 198	72 (12) 283		67 (28) 193	
78	*p48/p45*	68579 > 69721	380	E	83 (57) 439	74 (56) 392	69 (54) 396	75 (58) 379	63 (52) 377	91 (55) 372	72 (55) 394	90 (55) 372	64 (57) 389	73 (51) 373	95 (56) 372	68 (58) 389	103 (32) 187
79	*p12*	69751 > 70098	115	L	84 (50) 109	75 (48) 110	70 (44) 118	76 (24) 108	64 (36) 96	92 (39) 120	73 (55) 106	91 (39) 121	65 (38) 111	74 (41) 112	96 (42) 121	69 (44) 109	102 (13) 122
80	*bv/odv-c42*	70165 > 71286	373	L	85 (51) 380	76 (49) 379	71 (46) 396	77 (46) 379	66 (48) 366	93 (44) 372	74 (54) 373	92 (45) 371	66 (49) 374	75 (52) 369	97 (45) 370	70 (51) 374	101 (19) 361
81	*p6.9*	71306 > 71473	55	L	86 (74) 49	77 (63) 58	72 (50) 55	78 (56) 54	67 (63) 56	94 (72) 60	75 (67) 61	93 (72) 58	67 (65) 57	75b (74) 60	98 (74) 60	71 (63) 58	100 (44) 55
82	*lef-5*	71514 < 72266	251		87 (54) 242	78 (55) 240	73 (51) 247	79 (54) 240	69 (52) 247	95 (53) 245	76 (53) 251	94 (53) 268	68 (57) 240	76 (51) 247	99 (53) 266	72 (57) 245	99 (41) 265
83	*38K*	72198 > 73085	295	L	88 (50) 343	79 (52) 343	74 (54) 298	80 (50) 299	70 (50) 340	96 (51) 301	77 (53) 297	95 (51) 301	69 (53) 298	77 (52) 299	100 (52) 303	73 (54) 301	98 (40) 320
84	*pif-4*	73088 < 73576	162	L	89 (42) 161	80 (44) 162	75 (36) 166	81 (36) 162	71 (42) 161	97 (40) 157	78 (48) 161	96 (40) 157	70 (46) 161	79 (42) 159	101 (40) 157	74 (44) 161	96 (26) 173
85	*helicase 1*	73698 > 76952	1085	L	90 (34) 1131	81 (35) 1128	76 (34) 1138	82 (34) 1133	72 (33) 1124	98 (34) 1159	79 (35) 1156	97 (34) 1160	71 (36) 1122	80 (33) 1162	102 (34) 1158	75 (34) 1133	95 (22) 1221
86	*odv-e25*	76960 < 77601	213	L	91 (73) 213	82 (72) 213	77 (65) 217	83 (64) 214	74 (66) 214	99 (65) 220	81 (63) 217	98 (64) 220	72 (72) 213	81 (68) 217	103 (64) 219	76 (71) 214	94 (36) 228
87	*p18*	77624 < 78100	158	L	92 (44) 161	83 (42) 161	78 (35) 164	84 (40) 161	75 (49) 156	100 (40) 122	82 (49) 167	99 (37) 158	73 (44) 160	82 (37) 155	104 (38) 158	77 (48) 162	93 (28) 161
88	*p33*	78114 > 78878	254	L	93 (57) 251	84 (56) 250	79 (49) 267	85 (54) 252	76 (58) 250	101 (51) 251	83 (54) 252	100 (52) 252	74 (58) 251	83 (53) 251	105 (53) 251	78 (65) 251	92 (35) 259
89		78917 < 79177	86														
90	*chaB*	79174 < 79401	75	L						102 (29) 87	84 (31) 81	103 (31) 87		84 (38) 78	106 (27) 87		
91	*lef-4*	79408 < 80751	447		95 (51) 480	86 (51) 472	80 (46) 452	87 (49) 441	78 (46) 432	110 (43) 447	85 (48) 468	112 (43) 450	75 (50) 464	86 (46) 438	114 (41) 452	80 (50) 478	90 (32) 464
92	*vp39*	80789 < 81670	293	L	96 (40) 285	87 (40) 283	81 (42) 291	88 (37) 293	79 (42) 320	111 (44) 329	86 (46) 292	113 (44) 329	76 (42) 286	87 (45) 317	115 (44) 327	81 (46) 286	89 (29) 347
93	*odv-ec27*	81729 > 82583	284	L	97 (46) 288	88 (46) 287	82 (44) 282	89 (45) 287	80 (47) 287	112 (44) 288	87 (48) 298	114 (44) 288	77 (46) 284	88 (48) 284	116 (43) 288	82 (47) 286	144 (21) 290
94		83383 < 84462	359		99 (28) 394	90 (32) 370	83 (24) 355	92 (24) 403	82 (29) 340	113 (29) 373	88 (35) 381	116 (30) 372	78 (31) 401	89 (32) 340	117 (30) 404	83 (28) 383	
95		84552 > 84773	73	E, L	100 (40) 108	91 (45) 99	84 (18) 92	93 (33) 97	83 (32) 69	116 (46) 123	90 (42) 124	119 (46) 123	79 (29) 94	90 (38) 92	120 (46) 123	84 (36) 97	
96	*vp91*	84757 < 86457	567	L	101 (42) 665	92 (39) 618	85 (40) 591	94 (38) 712	84 (40) 533	118 (32) 741	91 (36) 656	121 (31) 736	80 (41) 656	92 (32) 557	122 (32) 751	85 (40) 648	83 (24) 847
97	*tlp20*	86411 > 86872	153	L	102 (27) 216	93 (28) 162	86 (31) 126	95 (32) 117	85 (23) 139	119 (21) 161	93 (29) 177	122 (21) 159	81 (31) 109	93 (23) 173	123 (21) 164	86 (30) 182	82 (16) 180

98		86841 > 87416	191	L	103 (68) 191	94 (67) 187	87 (62) 190	96 (64) 223	86 (58) 191	120 (56) 187	94 (56) 197	123 (56) 187	82 (67) 236	94 (56) 189	124 (57) 187	87 (64) 196	81 (43) 233
99	*gp41*	87413 > 88273	286	E, L	104 (64) 289	95 (62) 289	88 (47) 289	97 (59) 285	87 (46) 283	121 (48) 290	95 (58) 309	124 (48) 290	83 (61) 279	95 (57) 285	125 (48) 290	88 (64) 286	80 (32) 409
100		88301 > 88567	88	L	105 (30) 111	96 (30) 85	90 (29) 90	98 (28) 85	88 (23) 89	122 (29) 103	96 (26) 105	125 (28)103	85 (28) 84	96 (26)93	126 (32) 107	89 (28) 97	78 (21) 109
101	*vlf-1*	88545 > 89648	368	L	106 (49) 378	97 (47) 375	91 (48) 358	99 (50) 381	89 (45) 346	123 (48) 373	97 (49) 382	126 (48) 374	86 (46) 377	97 (48) 372	127 (50) 371	90 (48) 375	77 (31) 379
102		89658 > 89915	85	L	107 (62) 84	98 (63) 83	92 (58) 84	100 (58) 85	91 (58) 81	125 (62) 85	99 (61) 85	128 (62) 85	88 (63) 94	99 (61) 85	129 (64) 85	91 (69) 84	76 (30) 84
103		89928 > 90377	149	L	108 (52) 148	99 (55) 149	93 (45) 154	101 (53) 145	92 (37) 145	126 (38) 145	100 (48) 148	129 (38) 145	89 (55) 150	100 (42) 148	130 (38) 145	92 (50) 150	75 (17) 133
104		90771 > 90962	63														
105		90859 < 91272	137														63 (28) 155
106	*dna pol*	91293 < 94493	1056	E	111 (56) 1051	101 (55) 1045	94 (55) 1031	103 (58) 1021	93 (52) 979	132 (53) 1098	101 (58) 1145	134 (53) 1098	90 (58) 1044	101 (54) 1035	138 (52) 1094	93 (57) 1045	65 (33) 984
107	*desmoplakin*	94463 > 96463	666		112 (24) 718	102 (24) 720	95 (18) 539	104 (21) 751	94 (14) 651	133 (14) 661	102 (14) 735	135 (14) 661	91 (20) 763	102 (15) 603	139 (13) 663	94 (23) 741	66 (9) 808
108	*lef-3*	96438 < 97457	339	E, L	113 (18) 353	103 (20) 341	96 (18) 346	105 (14) 349	95 (17) 297	134 (18) 351	103 (21) 342	136 (18) 351	92 (19) 337	103 (21) 358	140 (17) 345	95 (20) 339	67 (12) 385
109	*odv-nc42*	97357 > 97800	105		114 (46) 126	104 (40) 127	97 (48) 125	106 (49) 125	96 (40) 128	135 (36) 120	104 (51) 129	137 (38) 136	93 (52) 125	104 (49) 129	141 (40) 136	96 (50) 125	68 (26) 192
110		97866 > 98342	148	E, L	115 (21) 168	105 (22) 167	98 (34) 172	107 (22) 179	97 (25) 194	136 (21) 171	105 (31) 177	138 (21) 171	94 (29) 188	105 (34) 159	142 (22) 171	97 (29) 172	
111	*iap-5*	98362 > 99132	256		116 (51) 275	106 (48) 274	99 (40) 279	108 (47) 271	98 (42) 281	137 (37) 285	106 (49) 283	139 (37) 284	95 (46) 270	106 (42) 263	143 835) 290	98 (45) 272	
112	*lef-9*	99116 > 100597	493		117 (69) 499	107 (70) 499	100 (65) 498	109 (71) 497	99 (66) 494	139 (67) 493	107 (70) 496	140 (67) 493	96 (68) 494	107 (67) 503	145 (66) 497	99 (68) 493	62 (53) 516
113	*fp*	100604 > 101044	146	L	118 (58) 161	108 (58) 153	101 (53) 147	110 (53) 151	100 (52) 138	140 (52) 147	108 (66) 148	141 (51) 147	97 (56) 156	109 (56) 145	146 (40) 70	100 (57) 150	61 (36) 214
114		101057 < 101545	162	L													
115	*DNA ligase*	101563 < 103167	534	E	120 (51) 570	110 (52) 576	103 (51) 547	112 (48) 560	101 (50) 523	141 (46) 527	110 (56) 565	142 (46) 527	99 (55) 557	110 (50) 579	148 (46) 552	102 (57) 568	
116		103236 > 103388	51														
117		103451 > 103663	70		122 (26) 66	112 (23) 68	104 (22) 63	117 (20) 66	103 (35) 66	143 (36) 66	112 (37) 70	144 (22) 66	101 (36) 72		150 (36) 66	104 (22) 71	
118	*fgf-2*	103615 < 104850	412		123 (33) 400	113 (34) 409	105 (19) 356	116 (30) 387	104 (23) 396	144 (21) 409	113 (33) 393	145 (22) 409	102 (31) 384	116 (25) 398	151 (22) 404	105 (31) 433	32 (12) 181
119	*alk-exo*	104912 > 107569	886		125 (43) 398	115 (43) 395	107 (41) 413	114 (46) 399	106 (47) 378	145 (39) 457	115 (48) 397	146 (39) 457	104 (46) 403	117 (41) 408	152 (40) 473	107 (50) 399	133 (31) 419
120	*helicase 2*				126 (46) 457	116 (46) 445	108 (35) 495	113 (48) 439	107 (48) 436	146 (46) 455	116 (47) 471	147 (46) 455	105 (46) 461	118 (51) 437	153 (48) 457	108 (49) 446	
121		107958 < 108806	282		130 (24) 138	118 (19) 125	109 (14) 281		108 (22) 138		117 (21) 301		106 (19) 376	119 (22) 317		109 (21) 335	
122	*lef-8*	108806 < 111319	837		131 (67) 873	119 (68) 865	110 (63) 857	121 (68) 827	109 (65) 838	148 (63) 859	118 (68) 883	149 (63) 859	107 (67) 860	121 (63) 849	155 (63) 860	110 (68) 880	50 (49) 876
123		111431 > 111739	102	E	132 (22) 131	120 (22) 114			110 (25) 114	165 (27) 118	120 (31) 156	163 (27) 118		122 (27) 120	169 (28) 118	111 (27) 111	
124		111763 < 112011	82		133 (27) 62					170 (16) 63	121 (39) 67		108 (20) 56			112 (31) 54	
125		111926 > 112327	133	E	134 (53) 133	121 (53) 133	111 (43) 179	122 (47) 133	112 (46) 137	171 (40) 139	122 (42) 136	169 (40) 139	109 (50) 133	127 (42) 165	173 (41) 139	113 (55) 133	53 (25) 139
126		112322 < 113362	347	L	135 (15) 354	122 (18) 314	113 (21) 302	123 (12) 322	113 (15) 198	172 (21) 378	124 (27) 371	170 (21) 378	110 (25) 295	128 (27) 316	174 (20) 373	114 (22) 323	
127		113436 < 113645	69	L					114 (24) 109	173 (26) 67	125 (28) 61	171 (26) 67		129 (28) 75	175 (26) 67	115 (23) 70	
128	*lef-10*	113626 > 113859	77	E	137 (34) 89	124 (32) 77		125 (36) 73		174 (42) 70	126 (40) 76	172 (42) 70	112 (38) 72		176 (40) 70		53a (20) 78
129	*vp1054*	113720 > 114754	344	E	138 (44) 332	125 (45) 331	115 (40) 324	126 (42) 328	115 (40) 311	175 (45) 323	127 (47) 331	173 (46) 323	113 (42) 330	130 (42) 320	177 (46) 323	116 (47) 330	54 (28) 365
130		114789 > 115022	77											132 (38) 52			
131	*fgf-3*	115040 > 116041	333	L	140 (29) 347	127 (33) 287	117 (25) 274	128 (24) 341	117 (19) 249	178 (22) 332	128 (26) 304	176 (21) 351	114 (33) 299	133 (24) 312	180 (23) 304	118 (34) 275	
132	*egt*	116583 < 117926	447	L	141 (48) 484	128 (47) 463	118 (36) 445	129 (45) 434	118 (48) 429		129 (51) 462		115 (51) 448			119 (52) 462	15 (38) 506
133	*me53*	117935 > 119056	373	L	143 (33) 303	129 (32) 303	119 (32) 303	130 (34) 297	120 (34) 308	180 (27) 325	131 (34) 309	178 (27) 325	116 (35) 310	134 (29) 289	182 (29) 304	120 (36) 303	139 (14) 449

The position and orientation (sense > or antisense <) of 133 ORFs are shown compared to 12 granulovirus homologs and AcMNPV. The presence of early or late promoter elements is indicated by E or L, respectively.
