# Supplementary material for: Genome of Epinotia aporema granulovirus (EpapGV), a polyorganotropic fast killing betabaculovirus with a novel thymidylate kinase gene
Source: BMC Genomics. 2012 Oct 11;13:548. doi: 10.1186/1471-2164-13-548 (PMC3496565; doi:10.1186/1471-2164-13-548)
Supplement: Additional file 2 — Multiple alignment of betabaculovirus alkaline exonuclease. This file shows the alignment of betabaculovirus alkaline exonuclease amino acid sequences. [file 1471-2164-13-548-S2.pdf]

```
AdorGV   --------------------------------------------------------MEDAPYKMAIADKRFNPIHEELAKRYSLQNFINNISNANQRISHDDIMVLEKCTRGQSDNVLWQLLRVNRSTAS-GSAVSEYDIDSIPAIRYGK
ChocGV   --------------------------------------------------------MAYELAV---SENVLNNQELLYAEKYCLQKYVKRLT-LTHRNTKEEIMALEHATRGQTNNMLWKILRINRTTASGGNT---FFEGSTPAMRYGI
CrleGV   --------------------------------------------------------MAYELAV---AEGNMTDLQLEFAKKFSLQNYVKTLT-LEHRNSKEEILKLEESTRGQSDNLLWKLLRVNRDTASGCSS---YCGNNNPAMQYGI
CpGV     --------------------------------------------------------MAYELAV---MEDVMSDVQLELARKYSMQNYVRTLT-LEHKNSREEILQLEEATRGQSDNLLWKLLRINRDTASGGSS---YCGGENVAMEYGL
EpapGV   --------------------------------------------------------MHEFNES-------LTAEQNRLADKYCLQNYVSNLT-LDYRNSKQEIFELEKATRGQGENELWKLLRINRTTAS--NSGHAFYGGENEAMRYGL
PhopGV   --------------------------------------------------------MSYEF-----FQDCMTPEQIMLAKKYCLQNYVRSLKSVDHRNTPEEIKQLEIATRGQADNILWKLLRVNRTTASKSTG---CFVEDSPAIQFGK
PiraGV   --------------------------------------------------------MAYELAV---SEGGMSDEQLGFAKKYMLQNYIKTLT-ESHRNTTEEIMELEKATRGQAKNVLWKLLRINRTTASGTSGG--FYGETTPAMKYGI
AgseGV   --------------------------------------------------------MDLSNCG-------FTDEQLKMAQKYCMENYVTKLK-PGHKNTKEEIFELE-ATRGQCKNNLWKLLRVNRTTAT-KSTGSSFYGES-VAMKYGI
HearGV   --------------MEQTRHTERVITMRFQNYPTYKYKRVSRDNSSVFLGTAYAMDDSSNNAQ---SNLAMNEYEQQLCDKYSYSNYVARLQ-PGHVNTREEIFALERATRGQSNNALWGVLRLNRKTASNRNGCSSFVSDKNEAIRYGN
PlxyGV   -----------------------------------------------------------MADK-------LTGYQCGLSDKYCLENYASRLT-AAHRNTKQEIYELEKATRGQSKNNLWKLLRINRKTAS-KST--YFSGDT-EAMRYGI
PsunGV   MRTESAVATTYCRNMDRTRLTERVITVRFQNYPTSNITAFFTINRLFHFEPAYTMDDNNSSSDPQLKKLAMNEYELQLCDRYSYSNYVARLK-PGHINTRKEIFELERATRGQNQNALWGVLRLNRKTASNLSGCSNFVSDNNEAIRYGI
SpliGV   ------------------------------------------------------MEEKSNSEF-------FTPEQYRLVLKYRYDLYITNLK-RGHYNTREEIFELEKATRGQSSNNLWRLLRINRTTAS-QSTCSAFNFET-EAIMYGN
XcGV     --------------MEQTRHTERVITMRFQNYPTYKYKRVSQDNNSVFLGPAYAMDDSSNNPQ---SKLAMNEYEQQLCDKYSYSNYVARLQ-PGHVNTREEIFALERATRGQSNNALWGVLRLNRKTASNRNGCSNFVSDKNEAIRYGN

AdorGV   IKEKALKNDNILISTIKDGIEDYTNKRVTETVLDCGMFLSSIGFYSASPDAYFKLEDGSMVVMEIKCPYSYKDDTIKDIRNRFNTNRARYRIPNTAFSINRHGEDIFVCVEAQNNHYRQMQLQMYATGALLAVYVVKFRDMPEVHFVKRD
ChocGV   CNETILKNNKAIINTITERIEKKLGKKVVENVLDCGLFITDIGLYSASPDGYFKLETGELVVLEIKCPYTYRNKTLEQIRREKNNTRGAYRVEHTALLVNRNGP-LHVTVTERNEHYRQMQSQIYVTNAIMAVYMVKFSDMPEIHFVEKN
CrleGV   KQEKILKEDKLIIDTVKDRIEKKLKKKIIEEVLDCGLFLSTIGLCSASPDAYFILENGELVVLEIKCPYNYRNESFKSVIQQLGSKRK--RIAHTALKRVSHDP-LIIHIEQRNNHYRQIQSQLYVTGAVMAVYLVKFSDKCDIHLVERN
CpGV     RNEKKLKKDNIVMGIIVERVEEKLNKKVVAQVLECGLFLSDIGLYSASPDAYFVLDSGELVVLEIKCPYTYRNDTFQSVLQQLDGRRS--RVARTALKRVSKQP-LILKVEQRNLHYRQMQSQLYVTGAVMAVYMVKFSDMPDIHFVERN
EpapGV   DNEKLLKQNAQLIDLVCARIEHKTKQKVVERVLDCGMFITELGLYSASPDAYFLLENNAMVVMEIKCPYTYRSETLFTIRDKFNN-RNRYRVPHTAFSVNRHGEYLNVLVEKNNDHYRQMQAQMYVTNAVFAVYMVKIGQDHEMHFVERD
PhopGV   NGERDLKQNTELIDLVCQKIEQKTGKKIVEKVLECGLFLSPIGLYSASPDAYFVLDDGNLVVLEIKCPYTYREDTLESVRHRMNSTRTRYRVAHTAFSVSRNGA-MNVRVEKQNDHYRQIQSQLYSTNAVLAVYMVKFVDFPEIHFVERD
PiraGV   DNEKLLKKNKIVMSIIREGVEKKLKKEIIEEVLDCGLFLSEIGLYSASPDAYFRLDNNDLVVLEIKCPYTYRNETLESIRLKMNSSRSRYRVPHTAFSVNRHDT-LHVAVEKRNDHYRQMQAQLYVTGAVMAVYMVKFSDMPEIHFVPRD
AgseGV   EQEEWIKQQKHIMDAVCEGIEGKLKKKVKERVLNCGLFLSPLGIYSASPDAYFVLENEELVVLEIKCPYTYKSDSMETIRRGFNN-RSRYRIPHTAFSINKNGP-IDVRVEKKNDHYRQMQAQLYITGAVLAVYLVKIGDVPEIHFVERD
HearGV   EQETVVKKNQLLMRTIIEKIEEKLSCDITETVLDCGMFISPIGLYSASPDAYFVNEQGQIIVLEIKCPYTYKDTNLESIRRNLNNNKARYRIKHTAFTINKQGP-IEVRVEKKNDHYRQLQSQMYVTGAVLGVYLVKIGDTEEVHFVERD
PlxyGV   EHEDLIKKNCIVMDVLCAEIENKLNASVRERVLECGLFLTELGLFSASPDGYFVLDDGRLVTMEIKCPFTYKDSSLEQIISGFNN-RSRYRIPNTAFSVNKTGP-VDIRVEKLNEHYRQMQWQMYVTGAIMAVYLVMIGDTPHVYFVDRD
PsunGV   NQEKVVKNNQLLMRAIKEKIEEKLDCVVTQTVLDCGMFISPIGLFSASPDAYFVDEKGQIVVLEIKCPYTYRNTNLCSIRNSFNN-RARYRIPNTAFSINKQGP-IDVRVEKKNDHYRQIQNQMYVTGAVLGVYLVKIGETEEVHFVERD
SpliGV   RNENVVKQNELLMRHIGEEIEHCLGKSIKEYVANCGMFISKMGIHSASPDAYYMLDDDNIVVVEIKCPYTYREKTLTSIRNSFNTNKSRYRITNTAFSINRSGP-PFVKVEEKNTHYRQMQAQMYVTGAVMAVYIVLIGQTPEVHFVERN
XcGV     EQETVVKKNQLLMRTIIEKIEEKLSCKITETVLDCGMFISPIGLYSASPDAYFVNEQGQIIVLEIKCPFTYKDTNLESIRRSLNNNKARYRIKHTAFTINKQGP-IEVRVEKKNDHYRQLQSQMYVSGAVLGVYLVKIGDTEEVHFVERD

AdorGV   SKFIQRVYNQEMNKLNRVVQSYKLQS-NMNTLAARMITYNRTNEFDTNSARLLAEAGMYSCDGNSVRCYICKSTYETADRSVESILSEHDN-LCN---VPKT--QAMHNSYLNIVDRINNLHQTN-LFLYEECQRLAQEGFVLIN--STT
ChocGV   EQYSKEMRHKELAKLQMYVNENKRSQ-IMIMESERLKTFTNT-NFKTGLAAALACDGMYYWCG-NVVCYFCCQQFEIVDKLLEEILEEHT--ECNKEGNISMGFKVTHPRYLNRFDRINSLRKTK--ENVVRIEELADKGFFYD---GSK
CrleGV   ESYIKELADKERFRLNMIIKENNRNK-KFTLEVHRLDSFKNS-GYDEEIVKQLAKNGLYCWCG-NVICFFCGQHFEINEKTIQQILNEHDNENCDKGDNCSM-TNVYNKRYLNIFDRINNLNESQ-TLSLSEIRDLAKKGYYND---GNK
CpGV     EAYIKGLADRERMKLEMYVKENKRNK-VMSIERERVNSFRGS-GYAREMVERLAREGLYCWCG-NVICYFCGQQFEVDNKSIEEVLGEHKVEQCDRTNNVSA-VRVHDNRYLNVFDRINNLHTVVTTLSSVERRQLAKKGYYYD---GNR
EpapGV   NEHINKMKKKELAEFKNYTNQNNSYR-CMIKEQSRYASFDS--AVEEQMRRKLAKNGLYNKHG-VITCYFCKSQFEINN-AIN-----HD---CGTKQDNVIYINVDNISYINERDRIDNLLNSAN-YCREQATALAKSKFYW---DGTQ
PhopGV   EKYIRDLAERERIRLKIYVDENKRNS-VMVSECERLKSFEGY-GYNEHLIKSLARDGLYCWCG-NVMCYFCQQQFEIIDKNADQILAEHN-LECTKLENIAI-STAAHPEYLNVHDRMHNLSESQ-LFNSVDCEKLAKEGFFIDQ--NNK
PiraGV   EKFIKELADRELIKLKMYANENKRSQ-IMVMEKERLKSFGGS-GHADKIARLLAKNGMYCWCG-NVICFFCKQQFEIIDKSIDIILEEHN-KECNRQENISM-VEVGHERYLNVFDRINNLLNTQ-KYSLIECEELAKKGYFYN---GSR
AgseGV   IKQIQELKDREEREFNNHVRDNSRRNNYLVLERNRKKTFPQNGAYSEEKSKLLARQGFYYNQG-CVVCYFCKSKYETST-PFLEMT--HS--NCD-KTDNISHVDIKFFNYINQKDRVNNLNKYCS-YDYQQCIELANQGFFYD-PSDNT
HearGV   EEMINDYANNEKSDLKEILSENAKHM-EFVMERNRLFSFYSLPNVKNENIKKLARDGFYYWNG-CVKCHFCQKHVELEN-DVDNILAQHV---CNSKHGNVRYADIKHRKYLTLQSRINSFIPLNI-DTT-LALELAKLNVFVA--DDNN
PlxyGV   EKLTKEIGERELNEFNRIVSDNNKKK-YLLMEVNRLKTFDS----ASTDSKLLAKNGFYQWYG-QIICYFCNLKYELDM-GVAAILDNHK--LCD-KTNNVKLISIAHPSYIKLQKRIDSITSTG------DRVELAQRGFFE---QNGV
PsunGV   EEMIKDFAKNEDSELKRVLSENAKHR-EFVMERNRLFSFYNSPNIKNDIVKKLARDGFYYWNG-CIKCHFCQKHVELEN-GLDNILAQHV---CNSKHGNVRYADIKHRNYLTLQSRINSFSRLNI-DST-LAQELAKLNVFVG--DDNN
SpliGV   ASVIQELEAKEKKDYTNYIINNERCR-MYVMEKNRRESFKDCELSK-AAILKLSRSGFYYHFG-KIICYFCRVQVELER-GVDNALLQHAP-DCN-KEGDLRHAIFKYPRYFSDKIREQSLDRHVS-D----KQTAAKNNLFC---DGSK
XcGV     EEMINDYANNEKRDLKEILSENAKHM-EFVMERNRLFSFYNLPNVKNENIKKLARDGFYYWNG-CVKCHFCQKHVELEN-DVDNILAQHV---CNSKHGNVRYADIKHRNYLTLQSRINSFIPLNI-DTT-LALELAKLNVFVA--DDNH

AdorGV   PELYCCGG------KDGHHQTCYKTQERKRIIESGS----
ChocGV   LVLYCCGGGKDLLGKERHTDQCKKKEN-------------
CrleGV   FVLYCCGG------EKLHSNECHKA---------------
CpGV     LVLYCCGG------ETTHKPECHKGQQ-------------
EpapGV   LRLYCCGQT------DLHAPNCS-----------------
PhopGV   LVLYCCGN------EQDHDNACTKATQN------------
PiraGV   LAFYCCGE------QETHKNDCYKIQQ-------------
AgseGV   LKLFCCGEI------DKHSQKCDKNGGETSN---------
HearGV   LKYYCCATTIS---DDKINQEC-IKATVINCPHSVDCDRY
PlxyGV   LSLFCCGG-------AVHNQDCRYEEM-------------
PsunGV   LKYYCCSTVIS---DDKIDQQC-IKDVVKNCAHSDDCDRY
SpliGV   IALYCCGNVIK---SDLAESPFDIKDLLDNIVHTIDCERF
XcGV     LKYYCCATTIS---DDKINQEC-IKATVINSPHSVDCDRY
```

Multiple alignment of Betabaculovirus Alkaline Exonuclease. Multiple alignment were carried out with ClustalX v.1.64 using default parameters. Columns shaded in light blue correspond to identities and columns shaded in light orange correspond to conservative changes.
