# Supplementary material for: Genome of Epinotia aporema granulovirus (EpapGV), a polyorganotropic fast killing betabaculovirus with a novel thymidylate kinase gene
Source: BMC Genomics. 2012 Oct 11;13:548. doi: 10.1186/1471-2164-13-548 (PMC3496565; doi:10.1186/1471-2164-13-548)
Supplement: Additional file 3 — Multiple alignment of betabaculovirus helicase 2. This file shows the alignment of betabaculovirus helicase 2 amino acid sequences. [file 1471-2164-13-548-S3.pdf]

```
AgseGV   ----------------MAEKRAIDELLDLVESNNKKQK-------------------------QS-SSR---------LYVPKKLNEEQQRIYDYVTR-VKKFAPIFVSGSAGTGKSALLIAIRDWCRAEKKVVWIVSYTNLAARNIEGK
ChocGV   -----------MLSASKNWPIKVFFTMDQSLCYTAAEEEK---------IYSEKND---TPINAKKRKIE--------IKYSYKLNKKQQQIFDILTEKEIYFKPVFVSGSAGTGKSALLTTLREHWQGLQKIVYVAAYTHLAARNVSGK
CrleGV   -------------------------MTETNSFCIVAEEK-----------NYTATN---VTKR--KRIER--------LQAPTTLNKEQKYLFDKVAD-THNFSPIFVTGSAGTGKSALLMTLRNYWRNQGKTVFVAAYTHLASRNIDGK
CpGV     -------------------------METDSFCIAAAEKRPINQSVIKGNNSGGDD---AVKRV-RRIER--------VDAPTKLNREQQLMFDRVAN-ARRFEPLFVSGSAGTGKSALLVALRNHWRERGKIVYVGAYTHLASRNIDGR
EpapGV   --------------MESVPLTRTIEPEETVESIAEHLKQY----------------------DEKFNTR---------LVKPVTLNQSQQKLFDYVVS-RQEFEPIFVSGSAGTGKSALLLALQKRWEDDKKIVMTVAYTHMAARNVNGT
PhopGV   -------------------------MNKIMIMPVPKRR---------------KTE---EGPNEDKFRHY--------LQTPKKLNASQQYLFDRLAR-AQKFDPIFVSGSAGTGKSALLIALRDHWLSQGKCVSVAAYTHLAARNIGGR
PiraGV   -------------------------MDQDLLSTVAENK------------KLIKMI---ATKYNNKQIKR--------LSAPSSLNAKQQHLFNFLVN-SDYFEPVFVSGSAGTGKSALLITLRNYWREQGKIVFVTAFTHLAARNIDGK
AdorGV   MVTTRLATKLKKERELSKAVLRAIKTMDASTCTSLDNMTTSVSTCTTSHDIVRSDSPIYEDMDTDSEVQLNCVTDESLRNKINNLNRQQRKLFDYVTS-VEKFTPIFVSGYAGTGKSHVLTVIRDYWKIQNKLVYTTAYTNAAARVVEGK
HearGV   -------------------------MKRQAVCNDKQE-------------------------DTKRICR---------LDAPTKLNEQQQQIFDYVTQ-RDSFEPIFVSGSAGTGKSALLKSLRTYWIDRKKVVWVVSFTNLAARNVDGQ
PlxyGV   -------------------------MKRCNSVDAEEG--------------------------GK-IMK---------LEVPLTLNEQQQKIYNYLTS-VDCFEPIFVSGSAGTGKSALLVTLTKAWTMKNMRVDVGTYTNLAARNVNGK
PsunGV   -------------------------MKRQAVCHEKQQ-------------------------DTKRICR---------LDAPAQLNEQQQRIFDYVTQ-RSSFEPIFVSGSAGTGKSALLKSLRTHWINHKKIVWVVSFTNLAARNVDGQ
SpliGV   -------------------------MKRSSLSASEEDGV-----------------------CANKLCKK--------ESAPTTLNKQQQQLFDYVTL-TKEFGPIFVSGSAGTGKSALLRALQSHWKN--KTIWVTTYTNLAARNVNGT
XcGV     -------------------------MKRQAVCNDKQE-------------------------DTKRICR---------LNAPTKLNEQQQQIFDYVTQ-RDSFEPIFVSGSAGTGKSALLKSLRTHWIDRKKVVWVVSFTNLAARNIDGQ

AgseGV   TIHSMFKFDFNLNI--SNYRINAPEFLIIDEISMVPAKMLNGIDAQLKRSTG-EDAAFGGVNTIVFGDLYQLPPVENRF--RQNFTLPPYHSHAWSDFRLFNLTINMRQSEELFIKALNLLRKGDASCQDFFNSKVIDQEPCLEEKINCT
ChocGV   TCHSLFGFDFDLNLVKT--YVGLPNYLIIDEISMIPEKMLDKIDSRLRQNSGNRYTPFGGVNVIVFGDLYQLPPVTK-----TSDYLPPYKADVWQCFRLFELTENMRQSETDFINNLNLLRIGDNTCLSYFNNMVLKTPQSLEEKLLYT
CrleGV   TCHSLFGFDFKLNLIDKK-NIGIPDYIILDEISMIPDKMLDGIDSRMRQVTREPQKPFGGVNTIVFGDLYQLPPIED----KRDMTLPPYSADIWSVFKLYELKHNMRQTEAEYIKNLNLMRSGEISCLKFFNTLVTKFSIGIEDLLVHT
CpGV     TCHSLFGFDFDLNLTDK--DVGVPNYIILDEISMIPDKMLDGIDSRMRQNTRNPHTPFGGVNVIVFGDLYQLPPVDKNNYKKREKVLPPYEADVWTEFKIYELGENMRQTEQEYIHNLNLLRLGDFSCLPYFNTLVMDFAPEIEEKVAHT
EpapGV   TCHSAFGFDFNLNLK-SYICNPVPNYLIIDEISMIPDKMLNGIDEKLRYNTG-VDKPFGGVNVIVFGDLYQLPPINDE---KKNNFKPPFYSRVWNSLSLYELTENMRQTEAEFIANLNMLRVGDIRCKKFFD-KLVTKPPLISESVKCT
PhopGV   TCHSLFGFDFDLNLIDR--CISIPHYLILDEISMIPEKMLDGIDARLRTTTRKYDQPFGGVNIIAFGDMYQLPPIDT------N--EPIYMSDVWNTFRLYELTENMRQSEHEFITNLNLLRVGDLNCLPYFNTLVMKQKPKIEDKLRCT
PiraGV   TCHSLFGFDFDMNITDK--RVGLPDYIIIDEISMIPEKMLDGIDLRMRQNSRNFELPFGGVNVVAFGDLYQLPPVNN----RINYTLPPYESDVWNLFKLYELTENMRHTEPEYIKNLNLLRVGDLRCLNYFDSLVSNRIPTVEEKVAFT
AdorGV   TCHSLFGFSFFGDHDAN-HTIKLPDCLIIDEISMLPKKMLEGIDQVLRNCRNATNDIFGGVNVIIFGDLYKTQPVVS-----YNNTLPCYKSELWHQFDLYMLNQNMRQTEEEFMTNLNLLRRGDAKCMNYFNSLEICTNVDDKKQLDYT
HearGV   TIHKQFGFDFKCNLNANNKTVGAPNYFILDEVSMVPAKMLQNIHTFFQQNTR-MDMPFGGVNTIIFGDLYQLPPISN----QQCYQLPPYCADIWKSLRLYHLTINMRQSESDFIDALNLLRVGEKKCLEFFNQKVMDHNITIEDQFECT
PlxyGV   TLHKLFGFDLKMELR-SNFCFNAPDYLIIDEISMVPDKMLAGIDERLQQAGL-NGIPFGGVNVVVFGDLFQLPPISN----DKDAAKPPYYASVWSSFKLYELTINMRQSEQEFIDALNKLRVGDLTCQKFFNKQVLKKPPSIAEKLQCT
PsunGV   TIHKQFGFDFKCNLNNGNRHAGAPNYFILDEVSMVPSKMLQNIHTFFQQNTR-LDLPFGGVNTIIFGDLYQLPPISN----QQCHQLPPYCADIWKSLSLYHLTMNMRQSESDFIEALNLLRVGDKKCLEFFDQKVMDHNVTIEDQFECT
SpliGV   TLHKQFKFNFKGEMN-TNACVGVPNYFIIDEISMVSSKMLQQIHECLQNNTQ-VDLPFGGVNTIVFGDLYQLPPIST----AKDKSLPPYHADVWKEFKLFELTENMRQNEKDFIDALNMLRIGDSRCQKFFDDKVLQKSPSVEEKLNTT
XcGV     TIHKQFGFDFKCNLNANNKNVGTPNYFILDEVSMVPAKMLQNIHTYFQQNTR-MDLPFGGVNTIIFGDLYQLPPISN----QQCYQLPPYCADIWKSLRLYHLTINMRQSESDFIDALNLLRVGDKKCLEFFNQKVMNHSITVQDQFECT

AgseGV   SLVSTHLEANHLNNICYEYVKSKSKDKK-EYQVKLIKTLEKRHTT--SMPYNKSQEEMIFKDNIKYCVGTRVMITLNVRDFVGENSFCNGDIGTIVQVS-DECLTIKREWDGTERKLTMHGVLFETE--DKNVLKTVYGLPITYGWAVTI
ChocGV   SLVSTHSEANALNNQCYNYNKSNE-----EFLCDISTHTTKWRRN--MLCFNVDQENLIFPQNLKVRKGTRVMITHTNDS------FCNGDLGIVESFKEHE-IYIRREHDDAIGVLRKRTLYFNTS--TKGMVKQVIGFPITYGWAVTI
CrleGV   SLVSTHREADDINMQCYIYNSEEKE----EIILKSTSSLVSWNFY--LNVFNTEQEKLIFRDSLKVCKGTRVMITHTTGD------FCNGDLGIIDNITDKG-VMITREHDNKNLFLSPICLNFYSN--IKNTVKQVTGLPLTYGWAITI
CpGV     SLVSTHDEANTINNECYNFVVNEA-----ETTLKCTTKLVPWSYK--VNVFNAQQERLIFKQELQVCPGTRVMVTHTTQH------FCNGDTGIIEYITDMG-VYIRREHDNSLQILGPITLHFSS---SRGYIKAVTGIPLNYGWAVTI
EpapGV   TLVPLNYKADIVNINCYKYIRGLNK-KAEEYTVKIEQHILRKTFENSRILFTKKQEEMIFQPGMKFCVGTRIMATQNING------FCNGDVGIVTEVDPEVGPLIKREHDGRSMWLTKATVMFSTD--DKQYVKMITGLPMRYGWGGTV
PhopGV   SLVSTHREADEINDQCYEAIADKES----ETVMESTHEMVPWSYK--ATVFNKDQEKVVFKDKLKVCIGTRVMITHSTQG------FCNGDMGTIKYISETAGLGIEREHDSVLSYLLPIKLNFNAN--TSGKMKQVTGLPITYGWAITI
PiraGV   SLVSTHKEADNINLQCYSYIADREQ----EIVHTCETKLLPWSHK--ITVYNADQERLIFKPNIKICPNTRIMVTHTTQH------FCNGDMGVIEYITELG-LYIRREHDNSLQLLTPIQLYFNTK--SKGVVKLVNGLPISYGWAITV
AdorGV   TLVSTNQEANNINYKCYNLFYRYP-----EMVYRLHSKQVHRDTC--DIIYNECQKMSIFRHYIKLKADTRVVVTFTTQN------FVCGDIGTVVAVNPNS-VVIRKDIDDKLYTLYNITLHFYPPGNVHRYIDAIIGLPINYAWAITM
HearGV   SLVPTHREADYINSKCYAHIKTISEEPVVEYILQLSVRRESRRLHS-MMVYGSGQEELIFRDKLKYCVGTRVMITHNLKGL----AFCNGDIGTVIAID-ERGVIVKRECDGLMEKINMVEIAFETD--KYTIVKVVTGIPICYAWAVTI
PlxyGV   SLVSTHKEADFNNNLCYNHIK---KNKD-EKTIELKEQYAQRFKH--DIVYNANQEKIIFKDGMKYCVGTRVMITQTVP----TTTLCNGDIGEIVSID-DEKLTFQRECDGQKFDVCQMTINFETS--NYHTMKCVTGLTICQAWAVTI
PsunGV   SLVPTHREADYINSKCYGHIKTISEEPVVEYILNLSVRREPRRMH--LMVYGSGQEELIFRDKLKYCVGTRVMITHNLKGL----SFCNGNIGTVMDID-ELGVLVKRECDGIQERINMVEIAFETE--KYNTVKVVTGLPMCYAWAVTI
SpliGV   SLVSTHNEANAINEQCYKRICIDKE----EHTVELSVEKTNRGRD--MIVFNQNQIDMIFKDKMKYCVGTRIMVTHNVAG-----VFCNGDVGEIVGIE-ERGVLIKREYDDRVLVLSMIELAFDDD--NWRTVTLITGLPMCYAWAITI
XcGV     SLVPTHREADYINSKCYAHIKSISEEPVVEYILQLSVRRESRRLHS-MMVYASGQEELIFRDKLKYCVGTRVMITHNLKGL----AFCNGDIGTVIAID-ERGVLVERECDGLMEKINMVEIAFETD--KYTIVKVVTGLPICYAWAVTI

AgseGV   HKAQGMTLRNLIVYPAKTFVAGQAYVALSRATHCDGLILVDPIPANSIKNMREVDKVYEEQIKFQFTEEEPEEETVLYKESIFN
ChocGV   HKAQGMTLKNLTVHPLRVFVPGQTYVALSRVTHSKGLKLAAPIPDSAFYDMSFVTLVYKSMPKIE-------------------
CrleGV   HKAQGMTIKNLIVFPKCLFAPGQAYVALSRCIHSNGLKLADPVPVRSVQKMNNITNVYKNMELFI-------------------
CpGV     HKAQGMTIKNLIVYPKCLFAPGQAYVALSRCTHSKGLKLSCRIPDEGVKDMSFITEVYNSMEKWC-------------------
EpapGV   HKNQGMTVINLIVNPDKVFVNGQAYVALSRVTHCSGLKLISPIPERHISVMNDVTKIYESMNKLVVE-----------------
PhopGV   HKAQGMTVKNLIVYPLRVFAPGQAYVALSRSTHSMGLRLVDRLPTNAVCSMFEVTEMYKKMRKFE-------------------
PiraGV   HKAQGMTVKNLTVYPACLFAPGQAYVALSRSTHSDGLNLVSAIPEKAISNMNFITKVYGAMEKFNV------------------
AdorGV   HKSQGLRIKKLIVKTDNVFAPGQLYSAISKAQNSSNLKLVNKITEKVIYNMDHIERVYNNMKNLIL------------------
HearGV   HKAQGMTVKNLIVHPVNIFAKGQTYVALSRVTHCEGLRLVNKIPVECVMKMSDVDKVYATQDLLDTYND-FEEEFEYDI-----
PlxyGV   HKAQGMTLKNLIVYPERTFVEGQAYVALSRVTHCDGLKLVNKIPDSSIIEMRETVQVYKNLERLSLE-----------------
PsunGV   HKAQGMTVKNLIVHPLNIFAKGQAYVALSRVTHCEGLRLVNKIPAECIMKMSDVDKVYTTQDLLTIDNDGFEEEFQFEFDI---
SpliGV   HKAQGMTVKSVIVHPECVFAKGQAYVALSRVTHFEGLKLVSPLPKKCIKKMKDVDKIYTSQPRLIL------------------
XcGV     HKAQGMTVKNLIVHPVNIFAKGQTYVALSRVTHCEGLRLVNKIPEECVMKMSDVDKVYATQDLLDTYND-FEEEFEYDI-----
```

Multiple alignment of Betabaculovirus Helicase 2. Multiple alignment were carried out with ClustalX v.1.64 using default parameters. Columns shaded in light blue correspond to identities and columns shaded in light orange correspond to conservative changes.
