# Supplementary material for: Genome of Epinotia aporema granulovirus (EpapGV), a polyorganotropic fast killing betabaculovirus with a novel thymidylate kinase gene
Source: BMC Genomics. 2012 Oct 11;13:548. doi: 10.1186/1471-2164-13-548 (PMC3496565; doi:10.1186/1471-2164-13-548)
Supplement: Additional file 4 — Characteristics of baculovirus genomes. This file lists the characteristics of baculovirus genomes, including virus name, genome size, number of ORFs and GeneBank accession number. [file 1471-2164-13-548-S4.pdf]

Genus	Name	Acronym	Size bp	Nº ORFs	Accesion Number	Reference
Alphabaculovirus NPVs Group I	*Antheraea pernyi* NPV-Z	AnpeNPV-Z	126.629	147	DQ486030	[1]
	Antheraea pernyi NPV-L2	AnpeNPV-L2	126.246	145	EF207986	[2]
	Anticarsia gemmatalis NPV D2	AgMNPV	132.239	152	DQ813662	[3]
	Autographa californica NPV C6	AcMNPV	133.894	156	L22858	[4]
	Bombyx mandarina NPV	BomaNPV	126.770	141	NC012672	Xu *et al*., 2009, unpublished
	Bombyx mori NPV T3	BmNPV	128.413	143	L33180	[5]
	Choristoneura fumiferana DEF NPV	CfDEFNPV	131.160	149	AY327402	[6]
	Choristoneura fumiferana MNPV	CfMNPV	129.593	146	AF512031	[7]
	Epiphyas postvittana NPV	EppoNPV	118.584	136	AY043265	[8]
	Hyphantria cunea NPV	HycuNPV	132.959	148	AP009046	[9]
	Maruca vitrata MNPV	MaviMNPV	111.953	126	EF125867	[10]
	Orgyia pseudotsugata MNPV	OpMNPV	131.995	152	U75930	[11]
	Plutella xylostella MNPV CL3	PlxyMNPV	134.417	152	DQ457003	[12]
	Rachiplusia ou MNPV	RoMNPV	131.526	149	AY145471	[13]
Alphabaculovirus NPVs Group II	*Adoxophyes honmai* NPV ADN001	AdhoNPV	113.220	125	AP006270	[14]
	Adoxophyes orana NPV	AdorNPV	111.724	121	EU591746	[15]
	Agrotis ipsilon MNPV	AgipMNPV	155.122	163	EU839994	[16]
	Agrotis segetum NPV	AgseNPV	147.544	153	DQ123841	[17]
	Apocheima cinerarium NPV	ApciNPV	123876	118	FJ914221	Zhang *et. al*, unpublished
	Chrysodeixis chalcites NPV	ChchNPV	149.622	151	AY864330	[18]
	Clanis bilineata NPV DZ1	ClbiNPV	135.454	139	DQ504428	[19]
	Ecotropis obliqua NPV A1	EcobNPV	131.204	126	DQ837165	[20]
	Euproctis pseudoconspersa NPV	EupsNPV	141.291	139	NC_012639	[21]
	Helicoverpa armigera MNPV	HearMNPV	154.196	162	EU730893	Tang *et al*., 2008, unpublished
	Helicoverpa armigera NPV C1	HearNPV C1	130.759	137	AF303045	[22]
	Helicoverpa armigera NPV G4	HearNPV G4	131.405	135	AF271059	[23]
	Helicoverpa armigera SNPV NNg1	HearSNPV	132.425	143	AP010907	[24]
	Helicoverpa zea SNPV	HzSNPV	130.869	139	AF334030	[25]
	Leucania separata NPV AH1	LeseNPV	168.041	169	AY394490	[26]
	Lymantria dispar NPV	LdMNPV	161.046	164	AF081810	[27]
	Lymantria xylina MNPV	LyxyMNPV	156.344	157	GQ202541	[28]
	Mamestra configurata NPV A	MacoNPV A-90-2	155.060	169	U59461	[29]
	Mamestra configurata NPV A	MacoNPV A-90-4	153656	168	AF539999	[30]
	Mamestra configurata NPV B	MacoNPV B	158.482	168	AY126275	[31]
	Orgyia leucostigma NPV CSF-77	OrleNPV	156.179	135	EU309041	Eveleigh *et al*., 2008, unpublished
	Spodoptera exigua NPV	SeMNPV	135.611	139	AF169823	[32]
	Spodoptera frugiperda MNPV	SfMNPV 19	132.565	141	EU258200	[33]
	Spodoptera frugiperda MNPV 3AP2	SfMNPV 3AP2	131.330	142	EF035042	[34]
	Spodoptera litura NPV G2	SpltMNPV	139.342	141	AF325155	[35]
	Spodoptera litura NPV II	SpltNPV II	148.634	147	EU780426	Li *et al*., 2008, unpublished
	Trichoplusia ni SNPV	TnSNPV	134.394	145	DQ017380	[36]

Genus	Name	Abbreviation	Size bp	Nº ORFs	Accesion Number	Reference
Betabaculovirus	*Adoxophyes orana* GV	AdorGV	99.657	119	AF547984	[37]
	Agrotis segetum GV	AgseGV	131.680	132	AY522332	Xiulian *et al.*, 2004, unpublished
	Choristoneura occidentalis GV	ChocGV	104.710	116	DQ333351	[38]
	Clostera anachoreta GV	ClanGV	101487	123	HQ116624	[39]
	Cryptophlebia leucotreta GV CV3	CrleGV	110.907	128	AY229987	[40]
	Cydia pomonella GV	CpGV	123.500	143	U53466	[41]
	Helicoverpa armigera GV	HearGV	169.794	179	EU255577	[42]
	Phthorimaea operculella GV	PhopGV	119.217	130	AF499596	Croizier *et al.*, 2002, unpublished
	Pieris rapae GV	PrGV	108.592	120	NC_013797	Zhang *et al.*, 2010, unpublished
	Plutella xylostella GV K1	PlxyGV K1	100.999	120	AF270937	[43]
	Pseudelatia unipuncta GV	PsunGV	176.677	183	EU678671	Li *et al.*, 2008, unpublished
	Spodoptera litura GV K1	SpltGV K1	124.121	136	DQ288858	Wang *et al.*, 2007, unpublished
	Xestia c-nigrum GV	XecnGV	178.733	181	AF162221	[44]
Gamma-baculovirus	*Neodiprion abietis* NPV	NeabNPV	84.264	93	DQ317692	[45]
	Neodiprion sertifer NPV	NeseNPV	86.462	90	AY430810	[46]
	Neodiprion lecontei NPV	NeleNPV	81.755	89	AY349019	[47]
Delta-baculovirus	*Culex nigripalpus* NPV	CuniNPV	108.252	109	AF403738	[48]

REFERENCES

1. Nie ZM, Zhang ZF, Wang D, He PA, Jiang CY, Song L, Chen F, Xu J, Yang L, Yu LL *et al*: **Complete sequence and organization of Antheraea pernyi nucleopolyhedrovirus, a dr-rich baculovirus**. *BMC Genomics* 2007, **8**:248.
2. Fan Q, Li S, Wang L, Zhang B, Ye B, Zhao Z, Cui L: **The genome sequence of the multinucleocapsid nucleopolyhedrovirus of the Chinese oak silkworm Antheraea pernyi**. *Virology* 2007, **366**(2):304-315.
3. Oliveira JV, Wolff JL, Garcia-Maruniak A, Ribeiro BM, de Castro ME, de Souza ML, Moscardi F, Maruniak JE, Zanotto PM: **Genome of the most widely used viral biopesticide: *Anticarsia gemmatalis* multiple nucleopolyhedrovirus**. *J Gen Virol* 2006, **87**(Pt 11):3233-3250.
4. Ayres MD, Howard SC, Kuzio J, Lopez-Ferber M, Possee RD: **The complete DNA sequence of Autographa californica nuclear polyhedrosis virus**. *Virology* 1994, **202**(2):586-605.
5. Gomi S, Majima K, Maeda S: **Sequence analysis of the genome of Bombyx mori nucleopolyhedrovirus**. *J Gen Virol* 1999, **80 (Pt 5)**:1323-1337.
6. Lauzon HA, Jamieson PB, Krell PJ, Arif BM: **Gene organization and sequencing of the Choristoneura fumiferana defective nucleopolyhedrovirus genome**. *J Gen Virol* 2005, **86**(Pt 4):945-961.
7. de Jong JG, Lauzon HA, Dominy C, Poloumienko A, Carstens EB, Arif BM, Krell PJ: **Analysis of the Choristoneura fumiferana nucleopolyhedrovirus genome**. *J Gen Virol* 2005, **86**(Pt 4):929-943.
8. Hyink O, Dellow RA, Olsen MJ, Caradoc-Davies KM, Drake K, Herniou EA, Cory JS, O'Reilly DR, Ward VK: **Whole genome analysis of the Epiphyas postvittana nucleopolyhedrovirus**. *J Gen Virol* 2002, **83**(Pt 4):957-971.
9. Ikeda M, Shikata M, Shirata N, Chaeychomsri S, Kobayashi M: **Gene organization and complete sequence of the Hyphantria cunea nucleopolyhedrovirus genome**. *J Gen Virol* 2006, **87**(Pt 9):2549-2562.
10. Chen YR, Wu CY, Lee ST, Wu YJ, Lo CF, Tsai MF, Wang CH: **Genomic and host range studies of Maruca vitrata nucleopolyhedrovirus**. *J Gen Virol* 2008, **89**(Pt 9):2315-2330.
11. Ahrens CH, Russell RL, Funk CJ, Evans JT, Harwood SH, Rohrmann GF: **The sequence of the Orgyia pseudotsugata multinucleocapsid nuclear polyhedrosis virus genome**. *Virology* 1997, **229**(2):381-399.
12. Harrison RL, Lynn DE: **Genomic sequence analysis of a nucleopolyhedrovirus isolated from the diamondback moth, Plutella xylostella**. *Virus Genes* 2007, **35**(3):857-873.

13. Harrison RL, Bonning BC: **Comparative analysis of the genomes of Rachiplusia ou and Autographa californica multiple nucleopolyhedroviruses**. *J Gen Virol* 2003, **84**(Pt 7):1827-1842.
14. Nakai M, Goto C, Kang W, Shikata M, Luque T, Kunimi Y: **Genome sequence and organization of a nucleopolyhedrovirus isolated from the smaller tea tortrix, Adoxophyes honmai**. *Virology* 2003, **316**(1):171-183.
15. Hilton S, Winstanley D: **Genomic sequence and biological characterization of a nucleopolyhedrovirus isolated from the summer fruit tortrix, Adoxophyes orana**. *J Gen Virol* 2008, **89**(Pt 11):2898-2908.
16. Harrison RL: **Genomic sequence analysis of the Illinois strain of the Agrotis ipsilon multiple nucleopolyhedrovirus**. *Virus Genes* 2009, **38**(1):155-170.
17. Jakubowska AK, Peters SA, Ziemnicka J, Vlak JM, van Oers MM: **Genome sequence of an enhancin gene-rich nucleopolyhedrovirus (NPV) from Agrotis segetum: collinearity with Spodoptera exigua multiple NPV**. *J Gen Virol* 2006, **87**(Pt 3):537-551.
18. van Oers MM, Abma-Henkens MH, Herniou EA, de Groot JC, Peters S, Vlak JM: **Genome sequence of Chrysodeixis chalcites nucleopolyhedrovirus, a baculovirus with two DNA photolyase genes**. *J Gen Virol* 2005, **86**(Pt 7):2069-2080.
19. Zhu SY, Yi JP, Shen WD, Wang LQ, He HG, Wang Y, Li B, Wang WB: **Genomic sequence, organization and characteristics of a new nucleopolyhedrovirus isolated from Clanis bilineata larva**. *BMC Genomics* 2009, **10**:91.
20. Ma XC, Shang JY, Yang ZN, Bao YY, Xiao Q, Zhang CX: **Genome sequence and organization of a nucleopolyhedrovirus that infects the tea looper caterpillar, Ectropis obliqua**. *Virology* 2007, **360**(1):235-246.
21. Tang XD, Xiao Q, Ma XC, Zhu ZR, Zhang CX: **Morphology and genome of Euproctis pseudoconspersa nucleopolyhedrovirus**. *Virus Genes* 2009, **38**(3):495-506.
22. Zhang CX, Ma XC, Guo ZJ: **Comparison of the complete genome sequence between C1 and G4 isolates of the Helicoverpa armigera single nucleocapsid nucleopolyhedrovirus**. *Virology* 2005, **333**(1):190-199.
23. Chen X, WF IJ, Tarchini R, Sun X, Sandbrink H, Wang H, Peters S, Zuidema D, Lankhorst RK, Vlak JM *et al*: **The sequence of the Helicoverpa armigera single nucleocapsid nucleopolyhedrovirus genome**. *J Gen Virol* 2001, **82**(Pt 1):241-257.
24. Ogembo JG, Caoili BL, Shikata M, Chaeychomsri S, Kobayashi M, Ikeda M: **Comparative genomic sequence analysis of novel Helicoverpa armigera nucleopolyhedrovirus (NPV) isolated from Kenya and three other previously sequenced Helicoverpa spp. NPVs**. *Virus Genes* 2009.
25. Chen X, Zhang WJ, Wong J, Chun G, Lu A, McCutchen BF, Presnail JK, Herrmann R, Dolan M, Tingey S *et al*: **Comparative analysis of the complete genome sequences of Helicoverpa zea and Helicoverpa armigera single-nucleocapsid nucleopolyhedroviruses**. *J Gen Virol* 2002, **83**(Pt 3):673-684.
26. Xiao H, Qi Y: **Genome sequence of Leucania seperata nucleopolyhedrovirus**. *Virus Genes* 2007, **35**(3):845-856.
27. Kuzio J, Pearson MN, Harwood SH, Funk CJ, Evans JT, Slavicek JM, Rohrmann GF: **Sequence and analysis of the genome of a baculovirus pathogenic for Lymantria dispar**. *Virology* 1999, **253**(1):17-34.
28. Nai YS, Wu CY, Wang TC, Chen YR, Lau WH, Lo CF, Tsai MF, Wang CH: **Genomic sequencing and analyses of Lymantria xylina multiple nucleopolyhedrovirus**. *BMC Genomics* 2010, **11**:116.
29. Li Q, Donly C, Li L, Willis LG, Theilmann DA, Erlandson M: **Sequence and organization of the Mamestra configurata nucleopolyhedrovirus genome**. *Virology* 2002, **294**(1):106-121.
30. Li L, Li Q, Willis LG, Erlandson M, Theilmann DA, Donly C: **Complete comparative genomic analysis of two field isolates of Mamestra configurata nucleopolyhedrovirus-A**. *J Gen Virol* 2005, **86**(Pt 1):91-105.
31. Li L, Donly C, Li Q, Willis LG, Keddie BA, Erlandson MA, Theilmann DA: **Identification and genomic analysis of a second species of nucleopolyhedrovirus isolated from Mamestra configurata**. *Virology* 2002, **297**(2):226-244.
32. Ijkel WFJ, van Strien EA, Heldens JG, Broer R, Zuidema D, Goldbach RW, Vlak JM: **Sequence and organization of the Spodoptera exigua multicapsid nucleopolyhedrovirus genome**. *J Gen Virol* 1999, **80 (Pt 12)**:3289-3304.
33. Wolff JL, Valicente FH, Martins R, Oliveira JV, Zanotto PM: **Analysis of the genome of Spodoptera frugiperda nucleopolyhedrovirus (SfMNPV-19) and of the high genomic heterogeneity in group II nucleopolyhedroviruses**. *J Gen Virol* 2008, **89**(Pt 5):1202-1211.
34. Harrison RL, Puttler B, Popham HJ: **Genomic sequence analysis of a fast-killing isolate of Spodoptera frugiperda multiple nucleopolyhedrovirus**. *J Gen Virol* 2008, **89**(Pt 3):775-790.
35. Pang Y, Yu J, Wang L, Hu X, Bao W, Li G, Chen C, Han H, Hu S, Yang H: **Sequence analysis of the Spodoptera litura multicapsid nucleopolyhedrovirus genome**. *Virology* 2001, **287**(2):391-404.

36. Willis LG, Seipp R, Stewart TM, Erlandson MA, Theilmann DA: **Sequence analysis of the complete genome of Trichoplusia ni single nucleopolyhedrovirus and the identification of a baculoviral photolyase gene**. *Virology* 2005, **338**(2):209-226.
37. Wormleaton S, Kuzio J, Winstanley D: **The complete sequence of the Adoxophyes orana granulovirus genome**. *Virology* 2003, **311**(2):350-365.
38. Escasa SR, Lauzon HA, Mathur AC, Krell PJ, Arif BM: **Sequence analysis of the Choristoneura occidentalis granulovirus genome**. *J Gen Virol* 2006, **87**(Pt 7):1917-1933.
39. Liang Z, Zhang X, Yin X, Cao S, Xu F: **Genomic sequencing and analysis of Clostera anachoreta granulovirus**. *Arch Virol* 2011, **156**:1185-1198.
40. Lange M, Jehle JA: **The genome of the Cryptophlebia leucotreta granulovirus**. *Virology* 2003, **317**(2):220-236.
41. Luque T, Finch R, Crook N, O'Reilly DR, Winstanley D: **The complete sequence of the Cydia pomonella granulovirus genome**. *J Gen Virol* 2001, **82**(Pt 10):2531-2547.
42. Harrison RL, Popham HJ: **Genomic sequence analysis of a granulovirus isolated from the Old World bollworm, Helicoverpa armigera**. *Virus Genes* 2008, **36**(3):565-581.
43. Hashimoto Y, Hayakawa T, Ueno Y, Fujita T, Sano Y, Matsumoto T: **Sequence analysis of the Plutella xylostella granulovirus genome**. *Virology* 2000, **275**(2):358-372.
44. Hayakawa T, Ko R, Okano K, Seong SI, Goto C, Maeda S: **Sequence analysis of the Xestia c-nigrum granulovirus genome**. *Virology* 1999, **262**(2):277-297.
45. Duffy SP, Young AM, Morin B, Lucarotti CJ, Koop BF, Levin DB: **Sequence analysis and organization of the Neodiprion abietis nucleopolyhedrovirus genome**. *J Virol* 2006, **80**(14):6952-6963.
46. Garcia-Maruniak A, Maruniak JE, Zanotto PM, Doumbouya AE, Liu JC, Merritt TM, Lanoie JS: **Sequence analysis of the genome of the Neodiprion sertifer nucleopolyhedrovirus**. *J Virol* 2004, **78**(13):7036-7051.
47. Lauzon HA, Lucarotti CJ, Krell PJ, Feng Q, Retnakaran A, Arif BM: **Sequence and organization of the Neodiprion lecontei nucleopolyhedrovirus genome**. *J Virol* 2004, **78**(13):7023-7035.
48. Afonso CL, Tulman ER, Lu Z, Balinsky CA, Moser BA, Becnel JJ, Rock DL, Kutish GF: **Genome sequence of a baculovirus pathogenic for Culex nigripalpus**. *J Virol* 2001, **75**(22):11157-11165.
